# Cationic gold nanoparticles elicit mitochondrial dysfunction: a multi-omics study

**DOI:** 10.1038/s41598-019-40579-6

**Published:** 2019-03-13

**Authors:** Audrey Gallud, Katharina Klöditz, Jimmy Ytterberg, Nataliya Östberg, Shintaro Katayama, Tiina Skoog, Vladimir Gogvadze, Yu-Zen Chen, Ding Xue, Sergio Moya, Jaime Ruiz, Didier Astruc, Roman Zubarev, Juha Kere, Bengt Fadeel

**Affiliations:** 1 Nanosafety & Nanomedicine Laboratory, Division of Molecular Toxicology, Institute of Environmental Medicine, Karolinska Institutet, 171 77 Stockholm, Sweden; 2 Department of Medical Biochemistry & Biophysics, Karolinska Institutet, 171 77 Stockholm, Sweden; 3 Department of Biosciences & Nutrition, Karolinska Institutet, 141 83 Huddinge, Sweden; 4 Division of Toxicology, Institute of Environmental Medicine, Karolinska Institutet, 171 77 Stockholm, Sweden; 5 Department of Molecular, Cellular, and Developmental Biology, University of Colorado, Boulder, CO 80309 USA; 6 CICbiomaGUNE, 20009 San Sebastian, Spain; 7 ISM, UMR CNRS 5255, Université de Bordeaux, 33405 Talence, France

## Abstract

Systems biology is increasingly being applied in nanosafety research for observing and predicting the biological perturbations inflicted by exposure to nanoparticles (NPs). In the present study, we used a combined transcriptomics and proteomics approach to assess the responses of human monocytic cells to Au-NPs of two different sizes with three different surface functional groups, *i*.*e*., alkyl ammonium bromide, alkyl sodium carboxylate, or poly(ethylene glycol) (PEG)-terminated Au-NPs. Cytotoxicity screening using THP-1 cells revealed a pronounced cytotoxicity for the ammonium-terminated Au-NPs, while no cell death was seen after exposure to the carboxylated or PEG-modified Au-NPs. Moreover, Au-NR3+ NPs, but not the Au-COOH NPs, were found to trigger dose-dependent lethality *in vivo* in the model organism, *Caenorhabditis elegans*. RNA sequencing combined with mass spectrometry-based proteomics predicted that the ammonium-modified Au-NPs elicited mitochondrial dysfunction. The latter results were validated by using an array of assays to monitor mitochondrial function. Au-NR3+ NPs were localized in mitochondria of THP-1 cells. Moreover, the cationic Au-NPs triggered autophagy in macrophage-like RFP-GFP-LC3 reporter cells, and cell death was aggravated upon inhibition of autophagy. Taken together, these studies have disclosed mitochondria-dependent effects of cationic Au-NPs resulting in the rapid demise of the cells.

## Introduction

Gold nanoparticles (Au-NPs) are attractive for different biomedical applications ranging from drug delivery to imaging due to their remarkable physicochemical properties^1,2^. Furthermore, early studies suggesting that Au-NPs are non-cytotoxic for human cells^3^ generated considerable enthusiasm for these materials. Subsequent investigations revealed that particle size and surface charge are important determinants of the cytotoxicity of Au-NPs^4–6^, though it was also noted that surface coating agents may account for at least some of the observed effects of these NPs^7^. Au-NPs were also shown to modulate autophagy in various cell types^8–10^, but the consequences of autophagy induction remain poorly understood. Autophagy (‘self-eating’) is a cell survival mechanism involved in cellular homeostasis; however, unrestrained autophagy may lead to cell death^11^. Indeed, while autophagy induction by NPs might be viewed as ‘an attempt to degrade what is perceived by the cell as foreign’^12^, cellular uptake of NPs can also trigger autophagy-dependent cell death under certain conditions^13,14^. Hence, a better understanding of the balance between autophagy and cell death following cellular exposure to NPs is required. It is also known that NPs may interact with proteins and other biomolecules leading to the formation of a bio-corona; the adsorption of proteins on the surface of Au-NPs may affect their uptake and other cellular responses^15,16^. However, protein binding of positively and negatively charged Au-NPs was shown to elicit different biological responses, *i*.*e*., cytokine secretion in monocytic cells^17^. Thus, the intrinsic physicochemical properties remain an important determinant of the biological effects of NPs.

Systems biology approaches based on computational modelling of systems-wide molecular changes at the gene or protein level are increasingly being applied in the field of nanosafety research for observing and predicting the biological perturbations inflicted by nanomaterials^18^. Transcriptomics refers to the study of the expression level of RNA molecules using either hybridization-based microarrays or next-generation sequencing (RNA-seq). For instance, microarray studies have shown that subtle changes in surface chemistry of Au-NPs may influence gene expression in human cell lines^19^. However, RNA-seq has several advantages over traditional microarray methods including a low background signal and the ability to quantify a large dynamic range of expression levels and this approach is being utilized to explore the impact of a variety of nanomaterials on cells in great detail^20–22^. Proteomics, in turn, provides an opportunity to study the full set of proteins expressed in a system, and recent mass spectrometry-based studies have shown that Au-NPs trigger endoplasmic reticulum (ER) stress in human chronic myelogenous leukemia cells^23^. Moreover, in a combined proteomics and metabolomics approach, Au-NPs were found to cause perturbations of multiple pathways in a human adenocarcinoma cell line^24^. Combining the information obtained from the transcriptome and proteome with cellular or physiological studies may yield further mechanistic insights into the effects of pollutants or chemicals^25^. Here we addressed the role of particle size and surface functionalization for cytotoxicity of Au-NPs using the human monocytic THP-1 cell line as a model. These cells are widely used to study the impact of NPs on immune-competent cells^17,26^. Studies were also conducted in *Caenorhabditis elegans*, a model organism that is increasingly used in toxicology^27^. To obtain a detailed understanding of the cellular effects of Au-NPs, we applied a combined transcriptomics and proteomics approach coupled with cell-based validation studies. Collectively, the results showed that ammonium-functionalized Au-NPs elicited mitochondrial dysfunction leading to cell death with features of both necrosis and apoptosis, and inhibition of autophagy was found to aggravate cell death suggesting that autophagy acts as a defence mechanism in this model.

## Results

### Ammonium-modified Au-NPs trigger dose-dependent cell death

**Figure 1:**
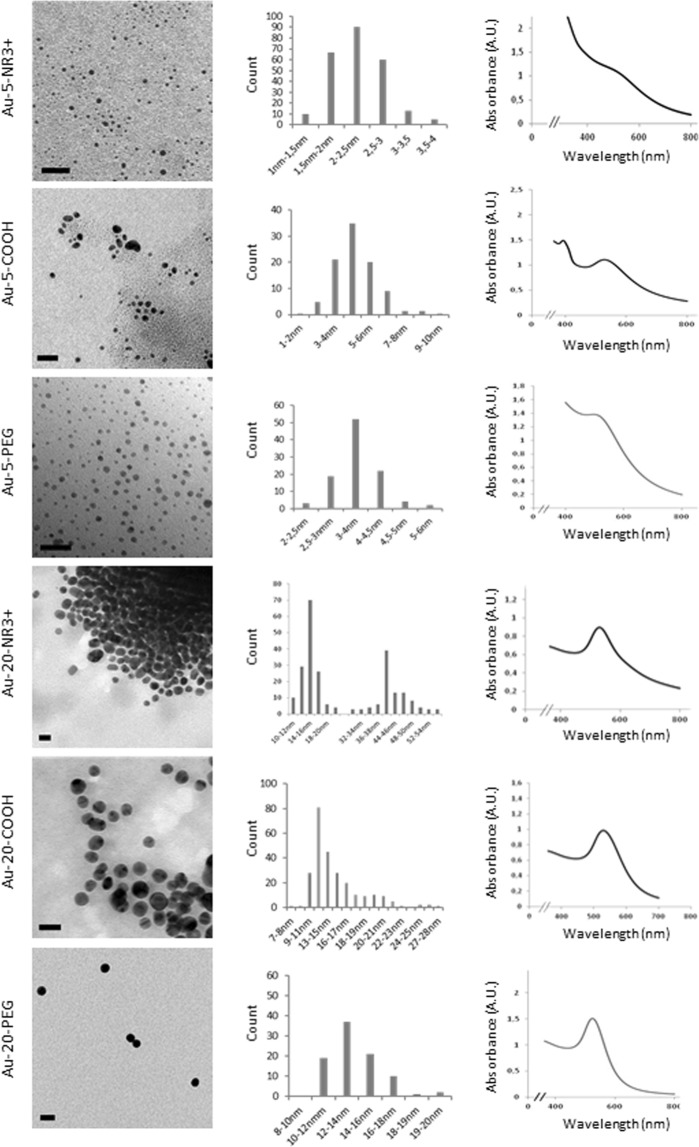
Physicochemical characterization of Au-NPs. TEM micrographs (scale bars: 20 nm) with size distribution histograms and UV-Vis spectra measurements in H_2_O are shown. For a summary of the DLS results in different media, see Supplementary Table S1.

Au-NPs (Au-COOH, Au-PEG, Au-NR3+) of two different core sizes were synthesized as described in the methods section. The primary particle sizes of the NPs were determined by transmission electron microscopy (TEM) whereas the size distribution profiles and zeta potentials of the NPs suspended in cell culture medium supplemented with serum were determined by dynamic light scattering (DLS) (Fig. 1, Supplementary Table S1). In addition, UV-vis spectra revealed the characteristic plasmon band of the Au-NPs (Fig. 1). It is noted that the hydrodynamic diameters of the Au-NR3 + NPs, and to a lesser extent of the Au-COOH NPs, increased when the NPs were dispersed in cell culture medium as compared to water, while no changes were recorded for the Au-PEG NPs (Supplementary Table S1). This is likely due to the agglomeration of the NPs in the presence of serum proteins. Moreover, the zeta potentials of the Au-NPs were ‘normalized’ upon dispersion in cell culture medium supplemented with serum, likely as a result of protein adsorption^28^.

Prior to biological testing, the particles were assessed for endotoxin content using the *Limulus* amebocyte lysate (LAL) chromogenic assay; however, Au-NPs were found to cause an interference with the assay (data not shown). To overcome this issue, the recently developed TNF-α expression test (TET) based on the activation of primary human macrophages was applied^29^. Briefly, human monocyte-derived macrophages, pre-incubated or not with polymyxin B, a specific LPS inhibitor, were exposed to Au-NPs, followed by the assessment of TNF-α secretion. LPS was used as a positive control for TNF-α secretion. The Au-NP samples were all found to be endotoxin-free (data not shown).

**Figure 2:**
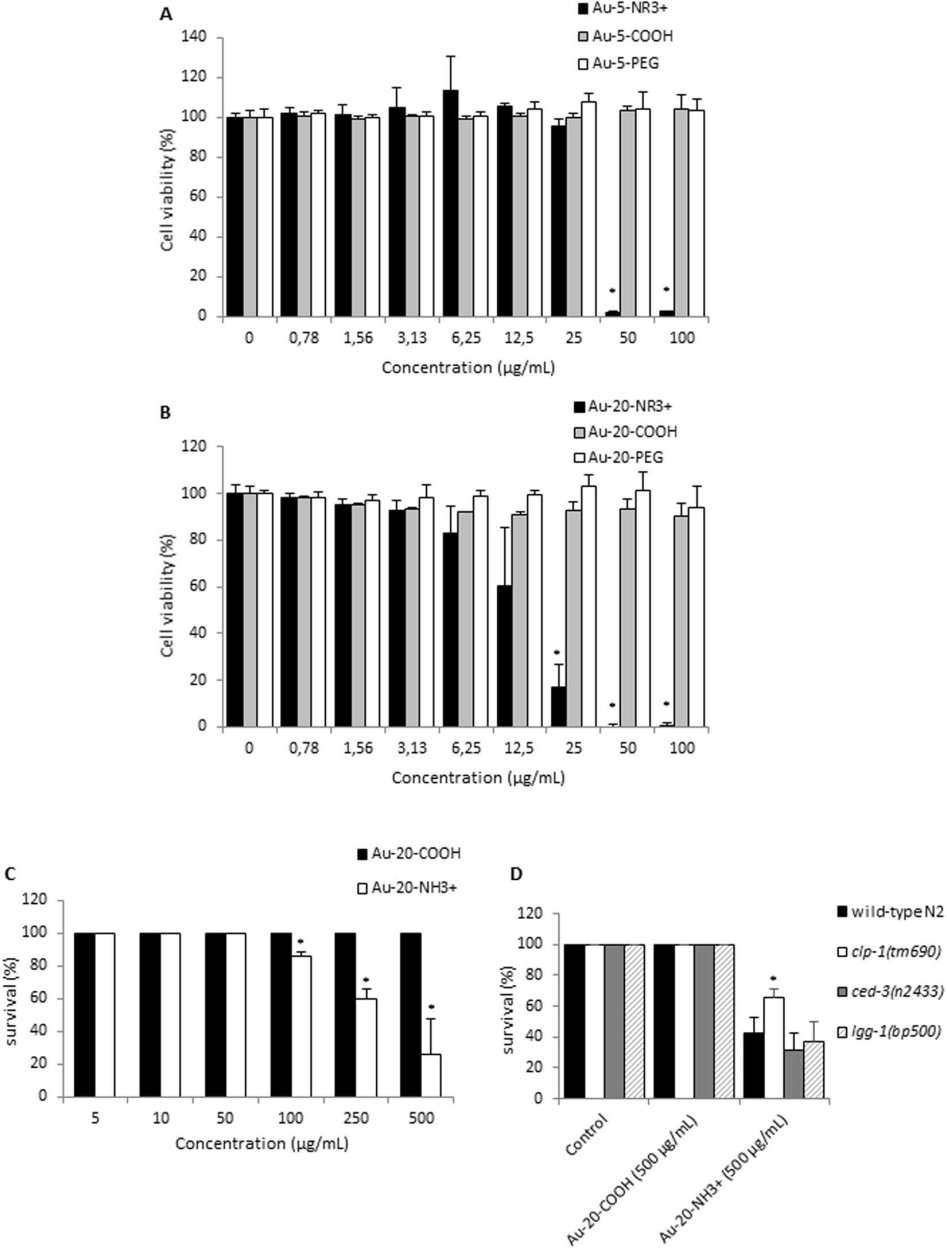
Cell viability and survival assessment. THP-1 cells were exposed for 24 h to Au-5 nm NPs (**A**) and Au-20 nm NPs (**B**). The percentage of living cells were determined by using the Alamar Blue assay. Data shown are mean values ± S.D. from 3 individual experiments each performed in triplicate. *p < 0.05 compared to control. (**C**) The survival rates of *C*. *elegans* N2 animals treated with Au-COOH NPs and Au-NR3+ NPs at the indicated concentrations for 24 h. The number of animals that survived was scored after treatment. 25 animals were scored for each concentration. Data shown are mean values ± S.D. from 3 individual experiments. (**D**) The effects of Au-NR3+ NPs (at 500 μg/mL) on animals defective for the selected cell death pathways (the *ced-3(n2433)* mutation blocks the apoptosis pathway, the *clp-1(tm690)* mutation blocks the necrosis pathway, and the *lgg-1(bp500)* mutations blocks the autophagy pathway). 25 animals were treated in each experiment. Data shown are mean values ± S.D. from 3 individual experiments. *P < 0.05.

For the evaluation of cytotoxicity, undifferentiated human THP-1 cells were exposed for 24 h to freshly dispersed Au-NPs at doses up to 100 µg/mL. Cell viability was determined by using the Alamar Blue assay; the amount of fluorescence is proportional to the number of living cells and corresponds to the metabolic activity of the cells. The particles did not interfere with the assay (data not shown). Dose-dependent cytotoxicity was observed for the ammonium-functionalized NPs while cell viability was not affected after exposure to the carboxylated or PEG-modified NPs (Fig. 2A,B). The concentrations required to trigger 50% cell death (EC_50_) were 34.8 µg/mL and 15.0 µg/mL for Au-5-NR3+ and Au-20-NR3+, respectively, indicating that the latter particles were more cytotoxic (Fig. 2A,B).

### RNA-sequencing reveals effects of Au-NPs on mitochondria

**Figure 3:**
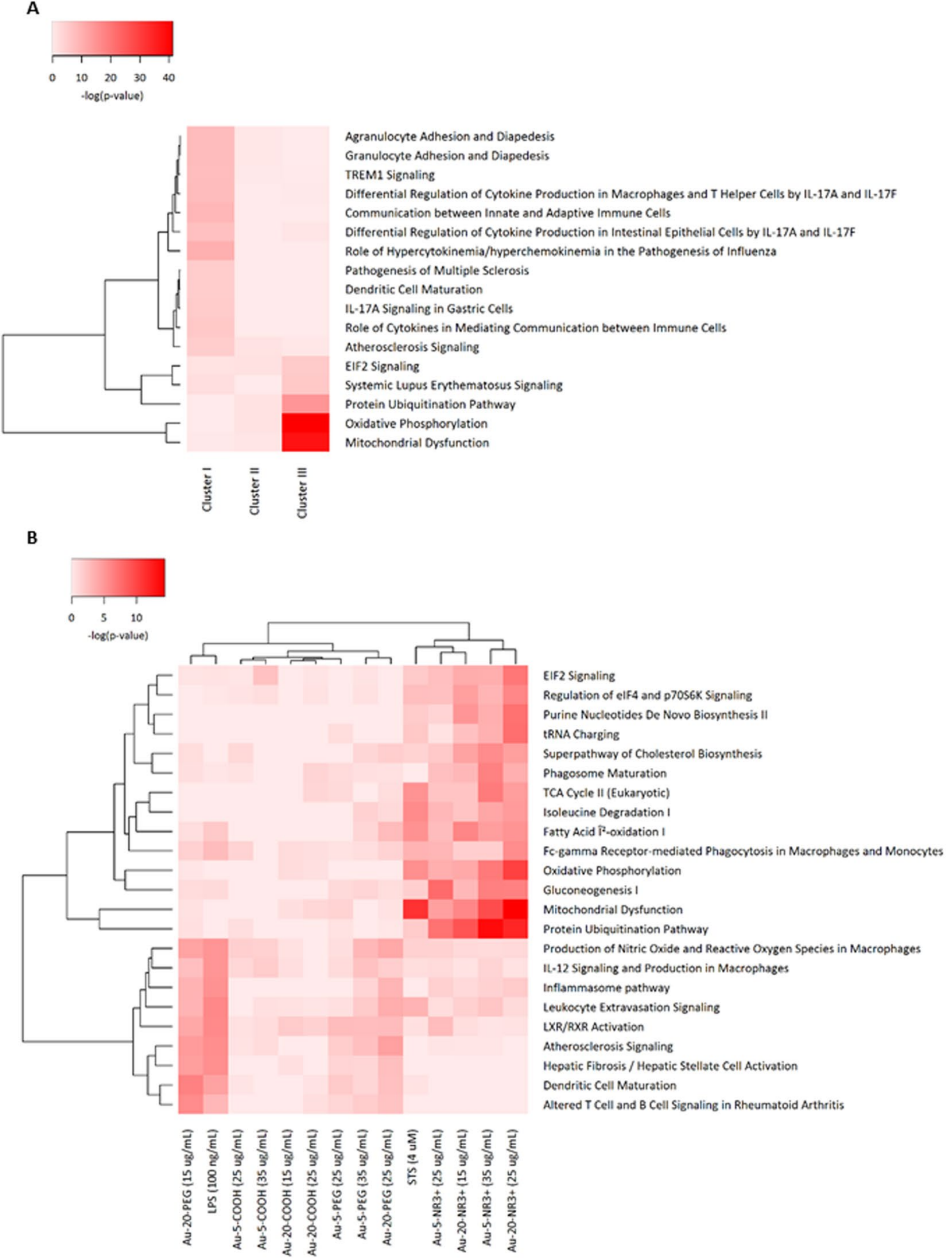
Pathway analysis of transcriptomics and proteomics data. (**A**) The transcriptomics data were submitted to pathway analysis using the Ingenuity Pathway Analysis (IPA) software. The heatmap shows the canonical pathways associated with the 3 clusters of DEGs (refer to Supplementary Fig. S1). Values are normalized scores of the -log(p-value) with *P* < 0.00001 for at least one cluster. (**B**) Pathway analysis of the proteomics data. Refer to Supplementary Fig. S3 for an overview of the proteomics results. The heatmap shows the canonical pathways associated with the various treatments. STS and LPS were used as positive controls for cell death and cell activation, respectively. Values are scores of the -log(p-value) with *P* < 0.00001 for at least one treatment. Data displayed in panel A and B were analyzed through the use of IPA (QIAGEN Inc., www.qiagenbioinformatics.com/products/ingenuity-pathway-analysis).

To gain a better understanding of the cellular impact of the NPs, we performed RNA-seq on THP-1 cells exposed to the entire panel of Au-NPs. We selected an early time-point (6 h) and sub-cytotoxic doses in order to capture early events and to avoid excessive cell death. Hence, RNA-seq was performed on total RNA samples from cells exposed to: (i) the 5 nm Au-NPs (-NR3+ /-COOH/-PEG) at a concentration of 27 µg/mL (corresponding to the combined EC_10_ dose for this set of NPs), (ii) the 20 nm Au-2NPs (-NR3+/-COOH/-PEG) at a concentration of 4 µg/mL (corresponding to the combined EC_10_ dose for this set of NPs), and (iii) all six Au-NPs at a concentration of 15 µg/mL (corresponding to the average EC_10_ dose). Following exposure, the cells were harvested and total RNA was extracted. RNA integrity number (RIN) values in the range of 8.90 to 10.0 were obtained for all samples, indicating that the sample preparation was successful and suitable for RNA-seq. Sequencing was performed using the Hi-Seq2000 Illumina platform and the data were aligned with the human genome UCSC hg19. Based on a multiple class comparison analysis, 777 differentially expressed genes (DEGs) over 13 classes of 37 qualified samples were identified as significantly fluctuated genes. Hierarchical clustering of the DEG expression profiles is shown in Supplementary Fig. S1. The activation scores indicate up- or downregulation of normalized expression levels of the genes. Notably, surface functionalization was the most significant factor followed by concentration and particle diameter (refer to colour coding at the top of the figure). Hence, major effects were observed for NR3+-modified Au-NPs, associated with a downregulation of DEGs at the highest concentration. Furthermore, three clusters of genes, designated I to III, were identified among the DEGs (Supplementary Fig. S1). To put these data into a biological context, gene ontology (GO) enrichment and pathway analysis was performed. The top-10 of the biological process terms, sorted by adjusted p-values, were found to be associated with inflammatory responses, protein rearrangement, and mitochondrial functions (Supplementary Table S2). The GO terms identified under cluster III displayed a strong adjusted p-value, reflecting the robustness of the data. Furthermore, pathway analysis of the transcriptomics data was performed with the Ingenuity Analysis Pathway (IPA) software^30,31^. A single core analysis was performed for each cluster (Supplementary Table S3), followed by a cross comparison analysis among the three clusters (Fig. 3A). On the basis of their p-values, the most significant pathways found to be associated with the DEGs dysregulated by the ammonium-modified Au-NPs in comparison to the COOH- or PEG-Au-NPs were “Mitochondrial Dysfunction” (p = 8.37E-39), “Oxidative Phosphorylation” (p = 5.02E-42), and “Protein Ubiquitination” (p = 1.51E-15).

**Figure 4:**
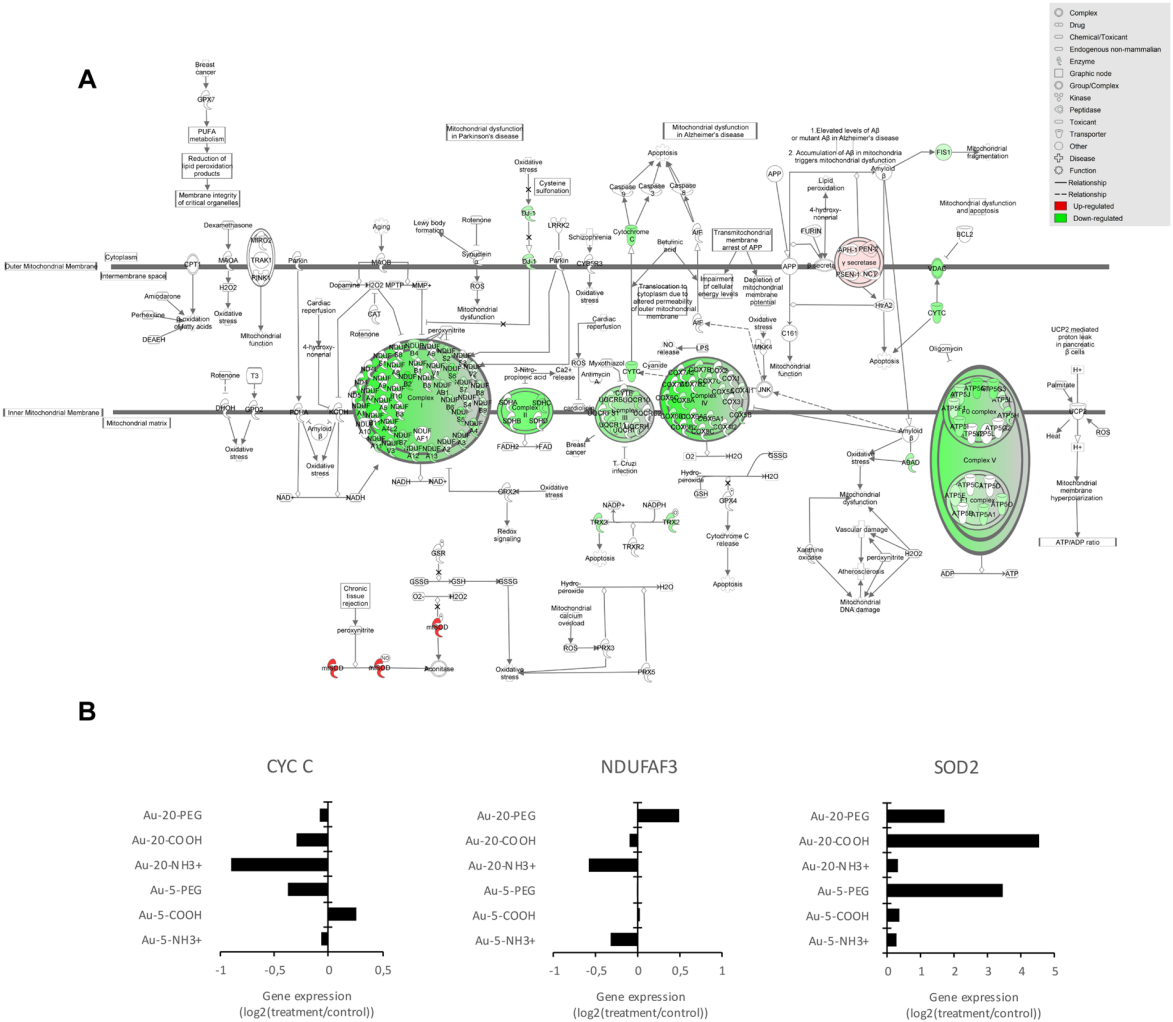
Dysregulation of the oxidative phosphorylation pathway. (**A**) IPA analysis showing dysregulated genes involved in oxidative phosphorylation and mitochondrial dysfunction pathways for Au-20-NH3+ NPs (15 µg/mL). The schematic figure illustrates complex I-V of the mitochondrial electron transport chain^90^. (green, downregulated; red, upregulated). Data were analyzed through the use of IPA (QIAGEN Inc., www.qiagenbioinformatics.com/products/ingenuity-pathway-analysis). (**B**) Selected examples of dysregulated genes belonging to the oxidative phosphorylation and mitochondrial dysfunction pathways. Cytochrome c (*CYT C*) is a small heme protein that transfers electrons between complexes III (Coenzyme Q–Cyt C reductase) and IV (Cyt C oxidase). *NDUFAF3* (NADH:ubiquinone oxidoreductase complex assembly factor 3) encodes a mitochondrial complex I assembly protein that interacts with complex I subunits. Mutations in this gene cause mitochondrial complex I deficiency, a fatal neonatal disorder. *SOD2* encodes mitochondrial superoxide dismutase. Refer to Supplementary Fig. S2 for further examples of dysregulated genes linked to oxidative phosphorylation.

The mitochondrial electron transport chain consists of a series of multiprotein complexes that oxidize reduced nicotinamide adenine dinucleotide (NADH) (complexes I, III, and IV) or reduced flavin adenine dinucleotide (FADH_2_) (complex II), membrane-bound mitochondrial glycerophosphate dehydrogenase, dihydroorotate dehydrogenase, electron transfer flavoprotein-ubiquinone oxidoreductase (ETF:QO) (complexes III and IV), transferring electrons to oxygen and pumping protons across the inner mitochondrial membrane. This creates an electrochemical proton gradient that drives the synthesis of adenosine triphosphate (ATP) by complex V in a process known as oxidative phosphorylation^32,33^. Upon further query of the transcriptomics data, we found that exposure to the Au-NR3+ NPs caused a pronounced downregulation of numerous genes encoding for subunits of the mitochondrial electron transport chain including cytochrome c (Figs 4A,B and S2). The only upregulated gene in this pathway was SOD2, encoding mitochondrial superoxide dismutase; SOD2 expression was affected more by carboxylated and PEG-modified NPs than by the ammonium-modified NPs (Fig. 4A,B). SOD2 binds the superoxide byproducts of oxidative phosphorylation and converts them to hydrogen peroxide and oxygen thereby protecting cells against cell death^34^.

### Proteomics analysis corroborates mitochondrial dysfunction

**Figure 5:**
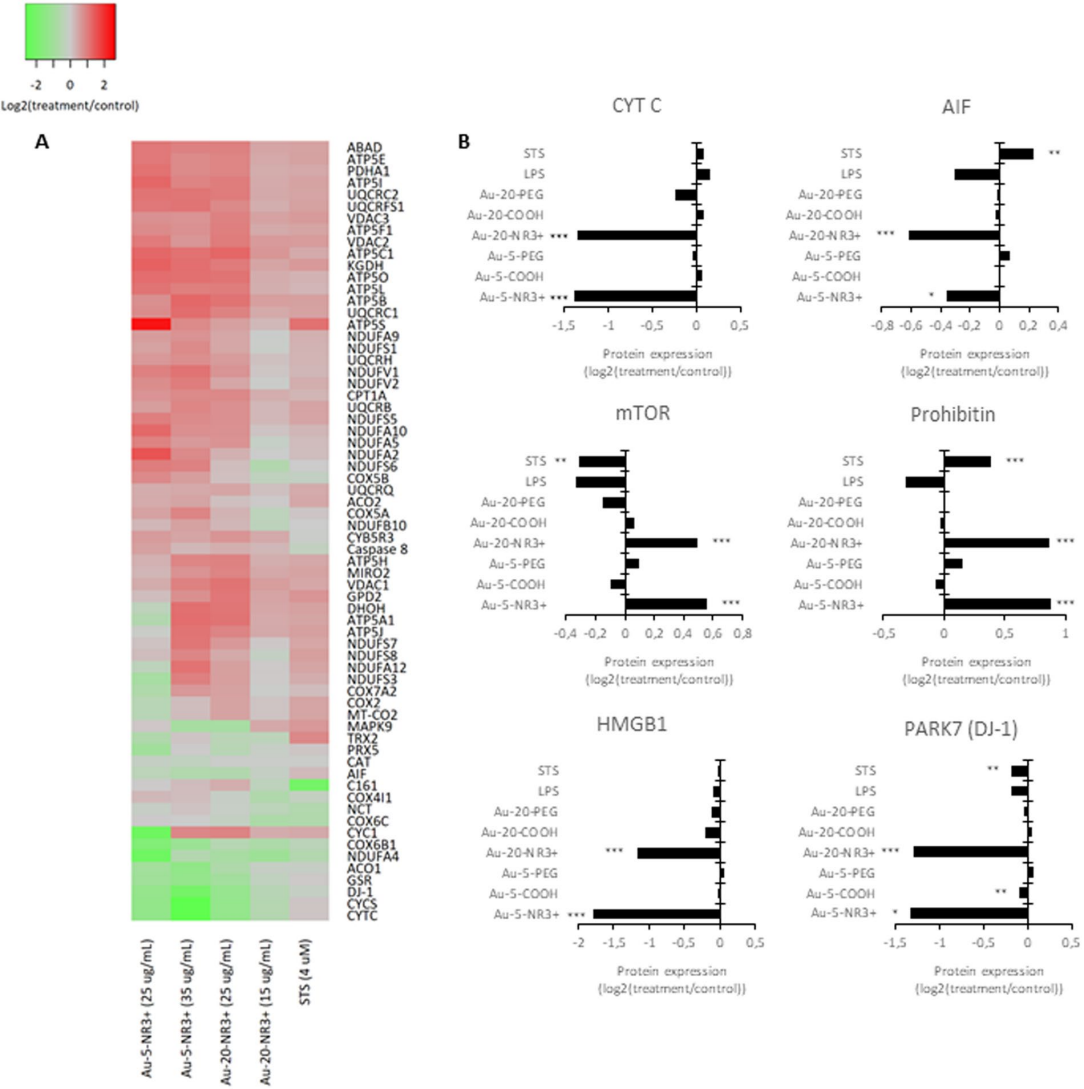
Dysregulation of mitochondrial dysfunction pathways. (**A**) Heatmap of the combined mitochondrial dysfunction and oxidative phosphorylation pathways based on the proteomics results for THP-1 cells exposed to Au-5-NR3+ NPs, Au-20-NR3+ NPs, and STS. The color scale represents the activation score obtained for the different treatments and normalized to control. (**B**) Histograms of selected proteins shown to be dysregulated in THP-1 cells exposed to Au-NPs (25 µg/mL), LPS (100 ng/mL), or STS (4 µM). Expression of cytochrome c, AIF, mTOR, prohibitin, HMGB1, and PARK7, *P ≤ 0.05, ** ≤ 0.01, and *** ≤ 0.001 (treatment *versus* control). For further details on these proteins and their roles in cell survival/cell death and autophagy/mitophagy, consult the main text.

Next, we performed proteomics analyses following acute exposure to Au-NPs. In contrast to the transcriptomics study, cells were exposed for 24 h at a dose that triggered 50% cell death (EC_50_) because the objective was to elucidate perturbations linked to cell death. Cells were thus exposed to: (i) the 5 nm Au-NPs (-NR3+/-COOH/-PEG) at a concentration of 35 µg/mL (corresponding to the combined EC_50_ dose for this set of NPs), (ii) the 20 nm Au-NPs (-NR3+/-COOH/-PEG) at a concentration of 15 µg/mL (corresponding to the combined EC_50_ dose for this set of NPs), or (iii) all six Au-NPs at a concentration of 25 µg/mL (corresponding to the average EC_50_ dose). Proteins were extracted and analyzed by mass spectrometry^35^. In total 3,998 proteins were identified and quantified by at least 2 peptides at <1% FDR. Hierarchical clustering showed that the ammonium-modified Au-NPs clustered together, distinct from the other NPs and the positive control for cell death, staurosporine (STS) (4 µM), as well as lipopolysaccharide (LPS) (100 ng/mL), a positive control for inflammation (Supplementary Fig. S3). Indeed, the most pronounced variations were observed for the ammonium-modified NPs with significant changes found in a large proportion of the quantified proteins (1,331 and 2,285 proteins for the 5 nm and 20 nm NPs, respectively). Pathway analysis of the significantly differentially expressed proteins was subsequently performed using the IPA software. The heatmap in Fig. 3B represents the canonical pathways associated with the different exposures. Notably, a close correspondence between the early changes observed by transcriptomics analysis at 6 h was found, as similar pathways were also affected at the protein level based on proteomics analysis at 24 h. Pathways linked to “Protein Ubiquitination” (p = 6.10^−8^ and 2.10^−14^ for Au-5-NH3+ at 25 or 35 µg/mL, respectively, and p = 7.10^−10^ and 1.10^−12^ for Au-20-NH3+ at 15 or 25 µg/mL, respectively), “Mitochondrial Dysfunction” (p = 3.10^−5^ and 3.10^−10^ for Au-5-NH3+ at 25 or 35 µg/mL, respectively, and p = 9.10^−7^ and 5.10^−15^ for Au-20-NH3 + at 15 or 25 µg/mL, respectively), “Oxidative Phosphorylation” (p = 2.10^−4^ and 2.10^−7^ for Au-5-NH3+ at 25 or 35 µg/mL, respectively, and p = 1.10^−4^ and 5.10^−11^ for Au-20-NH3+ at 15 or 25 µg/mL, respectively), and “Gluconeogenesis” (p = 2.10^−8^ and 3.10^−7^ for Au-5-NH3+ at 25 or 35 µg/mL, respectively, and p = 9.10^−4^ and 4.10^−7^ for Au-20-NH3+ at 15 or 25 µg/mL, respectively) were those mainly affected by the ammonium-modified NPs. To further highlight relevant protein changes, we focused on the two pathways, “Mitochondrial Dysfunction” and “Oxidative Phosphorylation” (Fig. 5A). Interestingly, the protein expression of several key proteins was affected mainly or only by the ammonium-modified Au-NPs and not by the other Au-NPs. Hence, both cytochrome c and apoptosis-inducing factor (AIF), two proteins involved in oxidative phosphorylation in mitochondria with additional roles in apoptosis following their release into the cytosol^36,37^, were found to be downregulated (Fig. 5B). Moreover, Au-5-NR3+ and Au-20-NR3+ NPs both upregulated mTOR (mechanistic target of rapamycin), a master regulator of cellular metabolism and autophagy^38^. Prohibitin, a recently identified mitophagy (*i*.*e*., mitochondrial autophagy) receptor involved in targeting mitochondria for autophagic degradation^39^ was also upregulated by Au-5-NR3+ and Au-20-NR3+ NPs, but not by the other Au-NPs, while PARK7/DJ-1, a transcription factor with pleiotropic functions including the maintenance of mitochondria by regulating their turnover by mitophagy^40^, was found to be significantly downregulated by Au-5/20-NR3+ NPs. HMGB1 (high mobility group box 1) was also significantly downregulated by these NPs, but not affected by any other treatments (Fig. 5B). HMGB1, a chromatin-associated nuclear protein with an auxiliary role as an extracellular cytokine^41^, is a key regulator of autophagy promoting cell survival by sustaining autophagy in response to cellular stress^42^. Taken together, multiple proteins known for their involvement in mitochondrial function and/or mitochondria-dependent regulation of cell survival and cell death were affected by the cationic Au-NPs.

### Mitochondrial dysfunction is validated by functional assays

**Figure 6:**
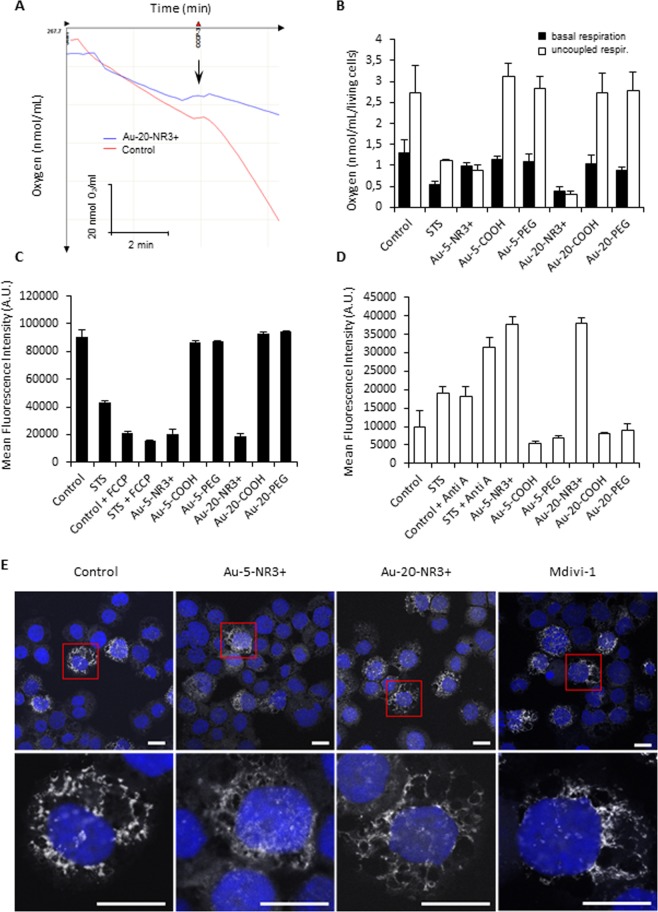
Cationic Au-NPs trigger mitochondrial dysfunction. (**A**) The oxygen consumption by THP-1 after exposure for 24 h to Au-20-NR3+ NPs (25 µg/mL) or culture medium alone (control). The uncoupling agent, CCCP (5 µM) was added in order to drop the mitochondrial membrane potential and maximally stimulate mitochondrial respiration. Representative results are shown. (**B**) The oxygen consumption by cells exposed to Au-5-NR3+ NPs or Au-20-NR3+ NPs for 24 h at 25 µg/mL or to STS (4 µM) was measured using an Oxygraph instrument in the presence or absence of CCCP. Data shown are the mean values ± S.D. (n = 3). (**C**) Mitochondrial membrane potential in cells exposed to Au-5-NR3+ NPs or Au-20-NR3+ NPs for 24 h at 25 µg/mL or to STS (4 µM) was quantified by flow cytometry using the TMRE assay. FCCP was used as positive control. Data shown are the mean values ± S.D. (n = 3). (**D**) Mitochondrial superoxide production in cells exposed to Au-5-NR3+ NPs or Au-20-NR3+ NPs for 24 h at 25 µg/mL was quantified by flow cytometry using the MitoSOX assay. Antimycin A (1 µM) was used as a positive control. Data shown are mean values ± SD (n = 3).** (E)** THP‐1 cells were exposed to Au‐5‐NR3+ NPs and Au‐NR3+ NPs for 4 h at 50 μg/mL and stained with MitoTracker Red. Mdivi‐1 (20 μM) was used as a positive control. Cell nuclei were counterstained with DAPI (blue) and samples were analyzed by confocal microscopy with a slice depth (Z stack) of 1 μm. Scale bars: 10 μm.

To validate the omics based predictions, we monitored mitochondrial function in THP-1 cells exposed to NPs. To this end, the impact of Au-NPs on mitochondrial respiration, mitochondrial membrane potential, and mitochondrial superoxide production was assessed (Fig. 6A–D). Au-5-NR3+ and Au-20-NR3+ NPs, but not the COOH- or PEG-modified Au-NPs, were found to significantly inhibit mitochondrial respiration. Figure 6A shows a representative example of the assessment of oxygen consumption rate and quantification of the results for the entire panel of NPs is reported in Fig. 6B. The uncoupling agent, carbonylcyanide-3-chlorophenylhydrazone (CCCP) (5 µM) was added in order to drop the mitochondrial membrane potential and maximally stimulate respiration. We also found that the two ammonium-modified Au-NPs caused a marked dissipation of the mitochondrial membrane potential in THP-1 cells (Fig. 6C). CCCP was included as a positive control. Similarly, mitochondrial superoxide production was observed for the ammonium-modified Au-NPs, regardless of particle diameter (Fig. 6D), while COOH- or PEG-modified Au-NPs did not decrease the mitochondrial membrane potential nor increase superoxide production. Antimycin (1 µM) was included as a positive control in these experiments. Our data showed that Au-NPs impair mitochondrial function in a surface-dependent manner in line with the multi-omics results.

Mitochondria are highly dynamic organelles that respond to cellular stress with fission and fusion^43^. Drp1 (dynamin-related protein 1), a member of the dynamin family of large GTPases, regulates cell survival and apoptosis by mediating mitochondrial fission^44^. To visualize the mitochondrial network of THP-1 cells, we performed confocal microscopy on cells stained with MitoTracker Red (Fig. 6E). Both Au-5-NR3+ and Au-20-NR3+ NPs (50 µg/mL) caused mitochondrial elongation suggesting a decrease in mitochondrial fission and an increase in mitochondrial fusion when compared to untreated cells. Mdivi-1 (20 µM), an inhibitor of Drp1^44^, was used as a positive control for mitochondrial fusion.

**Figure 7:**
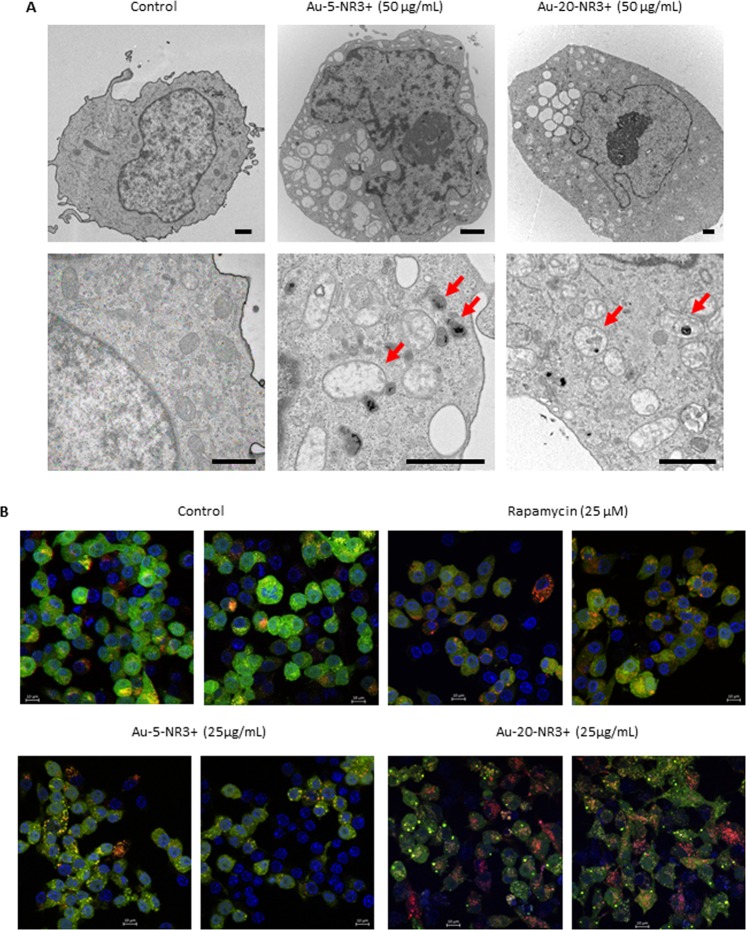
Cationic Au-NPs trigger morphological changes and autophagy. (**A**) THP-1 cells were exposed for 4 h with medium alone (control), Au-5-NR3+ NPs, or Au-20-NR3+ NPs (50 µg/mL). The results shown are representative images depicting the intramitochondrial localization of the NPs and ultrastructural changes including swollen and enlarged mitochondria (arrows). Au-COOH and Au-PEG NPs were not found in mitochondria (data not shown). Scale bars: 1 µm. (**B**) The RAW-Difluo mLC3 cell line was exposed to rapamycin (25 µM), Au-5-NR3+ NPs, or Au-20-NR3+ NPs at a non-cytotoxic dose (25 µg/mL) for 24 h. Untreated cells served as a negative control. Cells were then fixed and mounted on glass slides and cell nuclei were counterstained with DAPI (blue). GFP-LC3 (green) and RFP-LC3 (red) staining was investigated by confocal microscopy. Monitoring of autophagic flux in these reporter cells relies on the visualization of fluorescent puncta, loss of GFP signal (present in the autophagosomal membrane) in the lysosomal environment, and a concurrent increase of the RFP signal in autolysosomes^46^. Scale bars: 10 µm.

To obtain evidence for a direct interaction of the ammonium-modified Au-NPs with mitochondria in THP-1 cells, we exposed cells to Au-5-NR3+ NPs and Au-20-NR3+ NPs (50 ug/mL) and performed transmission electron microscopy (TEM). As shown in Fig. 7A, both NPs induced the formation of large vacuoles in the cytoplasm of THP-1 cells. This was not observed when cells were exposed to the COOH- or PEG-modified Au-NPs (data not shown). When viewed at higher magnification, evidence of ammonium-modified Au-NPs inside mitochondria was obtained; moreover, mitochondria in exposed THP-1 cells were swollen and enlarged (Fig. 7A), similar in appearance to the enlarged mitochondria seen in cells with a defective mitochondrial fission machinery^45^. To further evaluate the impact of these NPs on cells, and in order to corroborate the proteomics data, we used a murine macrophage-like reporter cell line expressing tandem fluorescent-tagged LC3 (*i*.*e*., RFP-GFP-LC3). These cells enable the monitoring of autophagic flux by detection of the appearance of green GFP-LC3 and/or red RFP-LC3 puncta by fluorescence microscopy^46^. We first evaluated cytotoxicity of Au-5-NR3+ NPs and Au-20-NR3+ NPs towards RAW264.7-LC3 cells and observed cytotoxicity at high concentrations (50 µg/mL and higher) (data not shown). We elected 25 µg/mL for further studies. As shown in Fig. 7B, Au-5-NR3+ NPs elicited a punctate green staining indicative of autophagosomes, while Au-20-NR3+ NPs triggered the formation of red-stained autophagolysosomes. The mTOR inhibitor, rapamycin (25 µM), was included as a positive control.

### Role of apoptosis versus autophagy in Au-NP exposed cells

**Figure 8:**
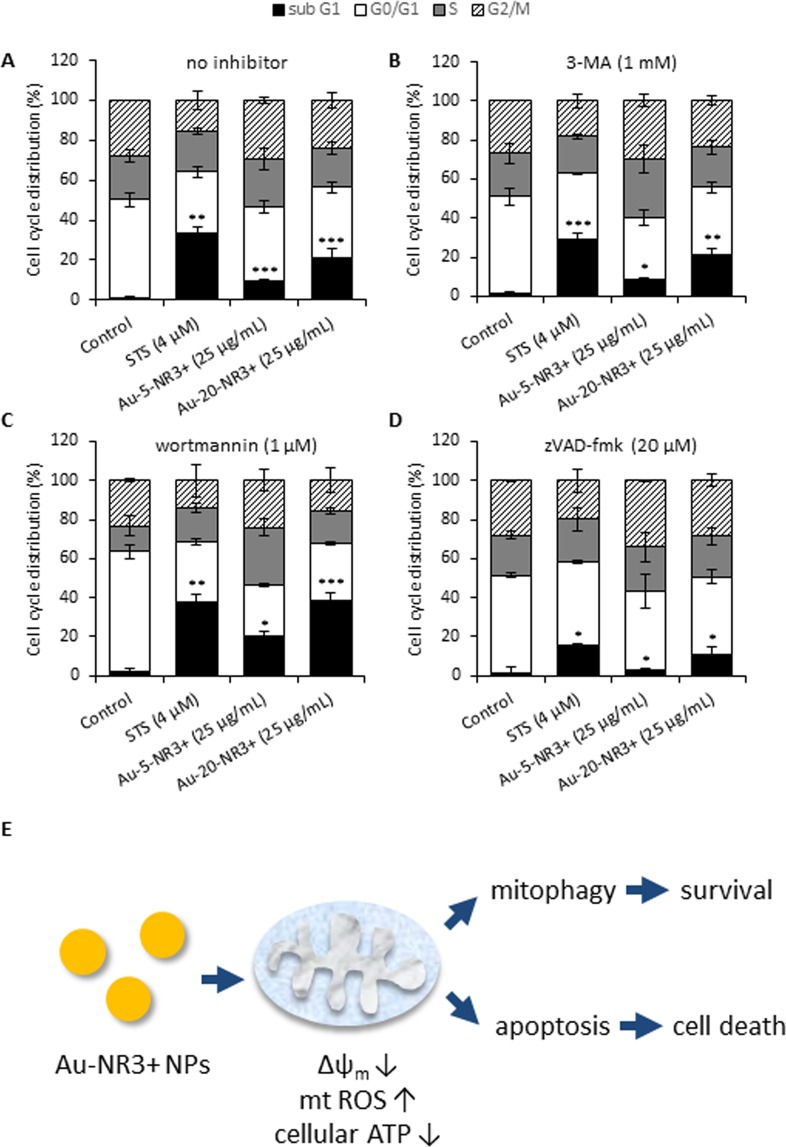
Cationic Au-NPs trigger apoptosis. (**A**) THP-1 cells were exposed for 24 h to Au-5-NR3+ NPs, or Au-20-NR3+ NPs (25 µg/mL). STS (4 µM) was used as positive control for apoptotic cell death. To determine the role of autophagy, cells were pre-incubated for 1 h with 3-MA (1 mM) (**B**) or wortmannin (1 µM) (**C**) prior to the exposure to NPs. Alternatively, cells were pre-incubated with the pan-caspase inhibitor, zVAD-fmk (20 µM) (**D**) to block caspase activity. Cells were then fixed and incubated with propidium iodide/RNAse A, and DNA content was analyzed by flow cytometry. Data are mean values of 3 experiments ± S.D. *P ≤ 0.05, ** ≤ 0.01, and *** ≤ 0.001 (treatment *versus* control). For corresponding results on caspase activation, refer to Supplementary Fig. S4. (**E**) Schematic diagram summarizing the impact of cationic Au-NPs on cells leading to mitochondrial dysfunction and apoptosis and a cytoprotective, autophagic response. At higher concentrations of Au-NPs and/or following prolonged exposure, cellular ATP levels are dissipated and the cells succumb to necrotic cell death (refer to Supplementary Fig. S5).

To test whether the ammonium-modified Au-NPs induced apoptosis, we performed flow cytometric analysis of cell cycle/cell death in THP-1 cells exposed to Au-5-NR3+ and Au-20-NR3+ NPs (25 µg/mL) in the presence or absence of autophagy/mitophagy or apoptosis inhibitors. STS (4 µM) was included as a positive control for apoptosis. The Au-20-NR3+ NPs, and to a lesser extent the Au-5-NR3+ NP, triggered apoptosis at 24 h, as evidenced by an increase in the sub-G1 population (Fig. 8A). Moreover, both ammonium-modified NPs elicited dose-dependent caspase-3 activation in THP-1 cells, as shown by the cleavage of the fluorescent substrate, DEVD-AMC (Supplementary Fig. S4). Interestingly, Au-NP-triggered apoptosis was accentuated following pretreatment of the cells with wortmannin (1 µM), an autophagy inhibitor, but not in cells pretreated with 3-MA (1 µM), also commonly used as an autophagy inhibitor (Fig. 8B,C). Additionally, apoptosis was inhibited in the presence of zVAD-fmk (20 µM), a pan-caspase inhibitor (Fig. 8D). In the absence of inhibitors, Au-5-NR3+ and Au-20-NR3+ NPs induced 9.5% and 21.3% apoptosis, respectively, while in the presence of wortmannin, apoptosis increased to 20.3% and 38.9%. In the presence of zVAD-fmk, apoptosis was reduced to 2.7% and 10.8%, respectively. These results suggest that the ammonium-modified Au-NPs triggered caspase-dependent apoptosis while the concomitant induction of autophagy/mitophagy fulfilled a cytoprotective function (Fig. 8E). We also asked whether pharmacological inhibitors of necroptosis (necrostatin-1) or ferroptosis (ferrostatin-1) would reduce cell death upon exposure to NR3+-modified Au-NPs, but these inhibitors were ineffective (data not shown). Similarly, CA-074-ME, a specific inhibitor of the lysosomal protease, cathepsin B, failed to block cell death (data not shown). The apoptosis studies referenced above were conducted at 25 µg/mL, corresponding to the average EC_50_ dose. To complement these experiments, we exposed THP-1 cells to Au-5-NR3+ NPs and Au-20-NR3+ NPs at 25 and 50 µg/mL. As shown in Supplementary Fig. S5A, ATP levels were drastically depleted in a dose- and time-dependent manner following exposure to 5 nm and 20 nm Au-NR3+ NPs. Furthermore, caspase-3 activation increased in a time-dependent manner in response to both Au-5-NR3+ NPs and Au-20-NR3+ NPs, but subsided again at later time-points (48 h) (Supplementary Fig. S5B,C), in line with the known requirement for ATP for those stimuli that act *via* mitochondria^47^. Hence, at higher doses and following prolonged exposure, ammonium-modified Au-NPs triggered necrotic cell death in the current model.

### Ammonium-modified Au-NPs cause loss of survival in *C*. *elegans*

The nematode *C*. *elegans* is ideally suited for *in vivo* screening of NPs^48^. To evaluate the potential *in vivo* impact of the Au-NPs, we tested the ammonium-modified Au-NPs in *C*. *elegans*. Biocompatibility was evaluated by studying the survival rate in young adult worms (*i*.*e*., 24 h post L4 larvae stage) exposed to Au-20-COOH NPs and Au-20-NR3+ NPs in the concentration range 0–500 μg/mL. We found that the Au-20-NR3+ NPs induced a significant, dose-dependent decrease in the survival of worms, compared to worms treated with the same concentrations of Au-20-COOH NPs. In particular, 500 μg/mL of Au-20-NR3+ resulted in more than 75% death of young adults, while exposure to the same concentration of Au-20-COOH NPs did not cause any lethality (Fig. 2C). Next, we assessed if the Au-NPs caused lethality in *C*. *elegans* through the apoptotic, necrotic, or autophagic cell death pathway. To this end, animals defective in the key proteases involved in apoptosis (*ced-3)* and necrosis (*clp-1*), or the autophagy gene (*lgg-1*)^49–51^, were exposed to 500 μg/mL of Au-20-NR3+ NPs or Au-20-COOH NPs. As shown in Fig. 2D, loss of *clp-1*, but not loss of *ced-3* or *lgg-1*, reduced lethality induced by Au-20-NR3+ NPs. These results confirmed that surface functionalization plays a role for the toxicity of Au-NPs.

## Discussion

In the current study, the importance of surface chemistry for cytotoxicity of Au-NPs was demonstrated. Thus, while COOH- or PEG-modified Au-NPs were non-cytotoxic, ammonium-modified Au-NPs elicited pronounced cytotoxicity in human immune-derived cells. The differences between the ammonium-modified Au-NPs *versus* COOH-modified Au-NPs were borne out *in vivo* by using *C*. *elegans* as a model. Furthermore, our combined transcriptomics and proteomics approach, coupled with multiple functional validation assays, provided a detailed picture of the underlying mechanism of toxicity, highlighting the crucial role of mitochondria, key regulators of survival and death of the cell^34,52^. Our results also showed that the Au-NR3+ NPs triggered autophagy, but this cytoprotective response was seemingly overwhelmed by the apoptotic response thereby ultimately leading to the demise of the cell. When higher concentrations of the NPs were used, cellular ATP levels were depleted and cells succumbed to necrosis. These results underscore the importance of surface chemistry and this may guide the design of safe nanomaterials for biomedical and other applications. Our results also pointed to a role of size insofar as the larger Au-NPs (20 nm) showed more pronounced effects when compared to the smaller ones (5 nm). It should be noted that the hydrodynamic diameters of the NPs changed as a result of the dispersion medium. Hence, our DLS measurements showed that the hydrodynamic diameters increased in cell culture medium supplemented with serum as opposed to H_2_O, likely as a result of protein adsorption which could lead to interparticle bridging, as shown previously for poly(acrylic acid)-coated Au-NPs^53^. Indeed, the latter study revealed that subtle changes in the diameters of NPs can have a pronounced effect on NP-protein interactions, which, in turn, may affect biological outcomes.

We observed that the zeta potentials of the Au-NPs were ‘normalized’ upon dispersion in cell culture medium supplemented with FBS. This phenomenon is well known and can be explained by the fact that most plasma proteins carry a net negative charge at physiological pH^28^. However, as we have clearly demonstrated in the present study, surface chemistry is a key determinant of the biological effects of Au-NPs, and this was confirmed both *in vitro* and *in vivo*. There could be different explanations for these observations. First, it has been shown that the adsorption of proteins on the surface of positively and negatively charged Au-NPs may elicit different biological responses^17^, and this could be due to the adsorption of different proteins and/or due to differences in the display of the adsorbed proteins on the surface of the NPs and whether or not the proteins are unfolded^54,55^. Second, it is also possible that the protein corona that is formed in the extracellular milieu due to the binding of serum proteins present in cell culture medium^15^ or proteins secreted from cells^56^ may ultimately be removed as the NPs enter the cells. Indeed, Bertoli *et al*. showed, using a combination of organelle separation and fluorescence labeling of the initial extracellular corona, that specific proteins present in the original protein corona are retained on the NPs until they congregate in lysosomes in the cell, at which point the proteins are degraded^57^. Thus, the intrinsic properties of the pristine NPs may come into play when the NPs are internalized, and this could explain the pronounced differences in cellular effects which we have observed for the ammonium-modified NPs *versus* carboxylated and PEG-modified NPs. It deserves to be mentioned that the molecular interactions of NPs within cells remain poorly understood, and though there has been a considerable emphasis on protein binding this does not exclude interactions with other biomolecules in the cell, such as lipids and nucleic acids (RNA, DNA).

RNA-sequencing is a powerful approach with which to assess genome-wide changes following exposure to toxicants including NPs^18^. In the present study, bioinformatics analysis of RNA-seq based transcriptomics data revealed that the “Mitochondrial Dysfunction”, “Oxidative Phosphorylation”, and “Protein Ubiquitination” pathways were the most significant pathways dysregulated by the ammonium-modified Au-NPs in comparison to the COOH- or PEG-modified Au-NPs. Similar changes were observed both for the 5 nm and 20 nm Au-NR3+ NPs. Moreover, these observations were highly concordant with our mass spectrometry-based proteomics analyses of THP-1 cells. Indeed, pathway analysis showed that pathways linked to “Protein Ubiquitination”, “Mitochondrial Dysfunction”, “Oxidative Phosphorylation”, and “Gluconeogenesis” were those mainly affected by the ammonium-modified Au-NPs. Previous studies of different nanomaterials have also provided evidence that the surface chemistry is an important determinant of cytotoxicity. For instance, using RNA-seq, we reported that low-dose exposure of primary human bronchial epithelial cells to cationic poly(amidoamine) (PAMAM) dendrimers triggered cell cycle arrest and a senescence-related gene signature while PAMAMs with a neutral surface charge did not elicit any significant changes in gene expression nor any cytotoxicity^20^. Furthermore, microarray studies have revealed that surface chemistry of Au-NPs plays an important role for gene expression changes in skin fibroblasts and prostate cancer cells^19^. Schaeublin *et al*. reported that mitochondrial stress and a decrease in mitochondrial membrane potential occurs in a human keratinocyte cell line following exposure to charged Au NPs, but not to neutral Au NPs^5^, and the degree of cyto- and genotoxicity of small (2 nm) Au-NPs towards human cervical carcinoma cells was found to depend on the hydrophobicity of the ligands attached on their surface^58^. Taken together, surface chemistry emerges as an important determinant of the cytotoxicity of Au-NPs and the present study has provided strong evidence for a role of mitochondria as key targets of ammonium-modified, but not COOH- or PEG-modified Au-NPs.

How NPs trigger cell death once they have gained entry into cells remains an area of active investigation^11^. Several studies have provided evidence for a role of lysosomes in cationic NP-induced cell death, with subsequent induction of apoptosis as a result of the release of cathepsins into the cytosol^59,60^. However, using a multi-omics approach, the present study has highlighted the crucial role of mitochondria for cell death elicited by ammonium-modified Au-NPs. In fact, Au-NR3+ NPs were found inside mitochondria of exposed cells, and these organelles were shown to undergo pronounced morphological changes suggestive of defective fission while functional assays revealed that mitochondrial function including ATP production was severely impaired, in line with the omics-based predictions which pointed to severe effects on mitochondrial oxidative phosphorylation. We could not demonstrate a role for lysosomal cathepsins; moreover, inhibition of endocytosis failed to prevent cell death triggered by ammonium-modified Au-NPs (data not shown). Taken together, this suggests that the endosomal-lysosomal pathway of NP uptake is not involved in this model and points instead to an alternative route of cell entry leading ultimately to mitochondrial uptake of the NPs with ensuing mitochondrial dysfunction. However, our results do not rule out the involvement of lysosomes, as we noted that THP-1 cells exposed to ammonium-modified Au-NPs displayed signs of autophagy and this was verified by using the autophagy reporter cell line, RAW-Difluo mLC3. The autophagosome delivers its contents to lysosomes followed by degradation of the delivered contents by lysosomal enzymes^61^. Therefore, in this sense, lysosomes are engaged in cells that are exposed to Au-NPs, although these organelles may not constitute the primary target in the case of ammonium-modified Au-NPs. Interestingly, recent studies have revealed that cationic NPs can enter cells in an energy-independent fashion, bypassing the traditional endocytosis route^62^. Using coarse-grained molecular dynamics simulations, model cell membranes were shown to generate nanoscale holes to assist the spontaneous translocation of cationic Au-NPs. After translocation, the Au-NPs moved freely in the ‘cytoplasm’ region. Furthermore, using whole-cell patch-clamp experiments to examine cell membrane porosity *via* measurement of the electrical conductance, Chen *et al*. could show that cationic NPs induced nanoscale defects such as a single hole or group of holes in the cell membrane in human cell lines^63^. One may also ask why the ammonium-modified Au-NPs were localized to mitochondria in the present study? Under stressful conditions, unfolded or misfolded proteins often clump together into high-molecular-weight aggregates, posing a threat to cellular homeostasis. Ruan *et al*. have shown that protein aggregates that are formed in cells accumulate at the surface of mitochondria and a fraction is then transported from the cell cytosol into mitochondria^64^. Intriguingly, blocking mitochondrial import, but not proteasome activity caused a marked delay in the degradation of aggregated proteins. One may speculate that ammonium-modified Au-NPs could be ‘sensed’ as misfolded proteins leading to their uptake into mitochondria. Further studies are needed to understand which signals are engaged in the mitochondrial sorting of protein aggregates^64^, and whether Au-NR3+ NPs, or clusters of such NPs, may act as protein ‘mimics’ thereby hijacking the same import mechanism. It is noteworthy that our multi-omics studies revealed a significant impact of the latter Au-NPs on the protein ubiquitination pathway which, in turn, is linked to proteasomal degradation (of proteins).

Autophagy is a cell survival mechanism; however, as discussed above, unrestrained autophagy may lead to cell death^11^. We noted that inhibition of autophagy using wortmannin, an inhibitor of phosphoinositide 3-kinases (PI3K), accentuated apoptosis in cells exposed to Au-5-NR3+ and Au-20-NR3+ NPs, while 3-MA, another PI3K inhibitor, had no effect. These findings may appear contradictory, as both 3-MA and wortmannin are widely used as autophagy inhibitors, based on their inhibitory effect on class III PI3K activity. However, Wu *et al*. recently provided a potential explanation in their study of 3-MA and wortmannin under nutrient-rich and nutrient-deprived conditions^65^. They demonstrated that wortmannin is able to suppress autophagy regardless of the nutrient status through a persistent inhibition of class III PI3K, while 3-MA promotes autophagic flux when used under nutrient-rich conditions, whereas it is still capable of suppressing starvation-induced autophagy. The actions of 3-MA were shown to be due to its differential effects on class I and class III PI3K^65^. With these observations in mind, our results suggest a cytoprotective role of autophagy in THP-1 cells exposed to ammonium-modified (cationic) Au-NPs. Ma *et al*. reported that Au-NPs with a negative surface charge were internalized into cells and eventually accumulated in lysosomes^9^. The NPs were found to cause autophagosome accumulation resulting from blockade of autophagy flux, rather than induction of autophagy. Ding *et al*. provided evidence, using negatively charged Au-NPs, that autophagy could protect human renal proximal tubular cells (HK-2) against apoptosis under normoxic conditions while, under hypoxic conditions, the NP-treated cells succumbed to apoptosis^66^. Based on electron microscopy imaging, the Au-NPs appeared to congregate in lysosomes in HK-2 cells. Furthermore, Ke *et al*. have shown that Au-NPs potentiated apoptotic responses to tumor necrosis factor-related apoptosis-inducing ligand (TRAIL) in non-small-cell lung cancer cells through Drp1-dependent mitochondrial fission^67^. The authors found that silencing of Drp-1 or inhibition of autophagy could alleviate apoptosis in cells co-exposed to Au-NPs and the pro-apoptotic factor, TRAIL. In contrast, our studies suggested a decrease in mitochondrial fission with an increase in mitochondrial fusion in THP-1 cells exposed to ammonium-modified Au-NPs when compared to untreated cells. The differences could be explained by a number of factors including the choice of cell model and whether or not the NPs translocated to the lysosomal compartment. Mitochondrial fission and fusion regulates mitochondrial number and morphology^43^. Impairment of mitochondrial fission-fusion processes can lead to the accumulation of damaged mitochondria which may result in mitophagy^68^. Prohibitin (PHB2) is a recently discovered mitophagy receptor of the inner mitochondrial membrane required for targeting of dysfunctional mitochondria for autophagic degradation^39^. It is pertinent that our proteomics analysis showed that PHB2 is upregulated in cells exposed to Au-NR3+ NPs. Hence, while doses equivalent to the average EC_50_ dose of the Au-NR3+ NPs elicited apoptosis with activation of caspase-3, as evidenced by DEVD-AMC cleavage, and a concomitant cytoprotective autophagy response (Fig. 8E), at higher doses these NPs triggered cell death by necrosis with a complete dissipation of cellular ATP levels and no caspase activation. Similarly, using a cervical carcinoma cell line as a model, Pan *et al*. reported that small Au-NPs (1.4 nm) triggered necrosis, not apoptosis, at high doses and the pan-caspase inhibitor, zVAD-fmk, failed to protect the cells from cell death^4^. Additionally, using *C*. *elegans* as a model, we noted that Au-NR3+ NPs, but not Au-COOH NPs, caused significant lethality, and experiments conducted at high doses of NPs showed that the Au-NR3+ NPs acted partially through the previously described CLP-1-dependent necrosis pathway^69^, while the nematode caspase, CED-3 was not involved.

In summary, our results have shown the importance of surface chemistry for the toxicity of Au-NPs. Indeed, while the ammonium-modified Au-NPs were cytotoxic and caused lethality *in vivo*, the COOH-modified Au-NPs were seemingly inert, as were the PEG-modified Au-NPs. We noted different modes of cell death depending upon the dose of administered NPs. The present study has revealed a remarkable concordance of the proteomics and transcriptomics results, and served to highlight the role of mitochondria as central regulators of toxicity resulting from exposure to the ammonium-modified Au-NPs.

## Materials and Methods

### Synthesis and characterization of Au-NPs

The strategy followed for the synthesis of the Au-NPs (Au-5 nm and Au-20 nm) consisted in growing the metallic NPs with the simultaneous attachment of self-assembled thiol monolayers on the growing nuclei in order to allow the surface reaction to take place during metal nucleation and growth. Briefly, Au-5 nm were prepared using reduction of Au^3+^ to Au0 with NaBH_4_ in the presence of bifunctional ligands of the type XRSH (X = COOH, N(CH_3_)_3_ or CH_3_)^70,71^ so that the surface was terminated with these functionalities. The ligands bound to the gold surface through their thiolate (RS end). Au-5-PEG were obtained by direct synthesis with controlled ratio of [HAuCl_4_]/[poly(ethylene glycol) methyl ether thiol (average 550 g/mol)]^72^. Au-20 were synthesized by reduction of of Au3+ to Au0 with Na3citrate which yielded Au NPs of core size >10 nm. The citrate reduction process involved hot gold chloride and sodium citrate as reactants. In this reaction, the citrate molecules acted as both reducing and stabilizing agents, allowing for the formation of the colloidal gold. The bifunctional ligands of the type XRSH (X = COOH, N(CH_3_)_3_ and CH_3_)^73–75^ were used in order to replace the citrate ligands on the NP surface. Poly (ethylene glycol) methyl ether thiol (average 550 g/mol) were the bifunctional ligands used in the synthesis of the Au-20-PEG^76^. TEM images of the NPs were obtained with a JEOL JEM-2100F UHR 200 kV instrument. For UV-vis and dynamic light scattering (DLS) measurements, fresh suspensions with a concentration of 0.1 mg/mL were prepared. UV-visible measurements were performed on a UV-vis spectrometer Lambda 750 (Perkin Elmer). The range of acquisition was 300 nm to 800 nm and the interval was set at 0.5 nm or 1 nm. UVWinLab software was used for data processing. DLS and zeta potential measurements were performed on a Zetasizer Nano ZS model ZEN3600 from Malvern using a 632.8 nm laser. The angle of detection was set at 173° and the temperature was maintained at 25 °C throughout analysis. Zetasizer v.6.32 software was used for data processing.

### Endotoxin assessment

The TNF-α expression test (TET) was used for assessment of endotoxin content in NP samples^29^. Briefly, primary human monocyte-derived macrophages (HMDMs) were obtained from human monocytes isolated from healthy donors by using a Lymphoprep density gradient (Axis-Shield, Oslo, Norway) as described previously^77^. The samples were completely anonymized and the Ethical Committee for Human Studies in Stockholm has previously issued a statement that no specific permit is required for *in vitro* studies of nanomaterials using cells derived from human donors since the data cannot be traced back to the individual blood donors (see: 2006/900-31/3; decision 2006/3:8). Cells were exposed to 5 µg/mL Au-NPs in culture medium supplemented or not with 10 µM polymyxin B (Sigma-Aldrich). LPS (100 ng/mL) (Sigma-Aldrich) was included as a positive control. Following incubation, supernatants were collected and the levels of TNF-α were monitored by ELISA (Mabtech, Nacka Strand, Sweden) according to the manufacturer’s instructions.

### Human and murine cell culture

The human monocytic THP-1 cell line was purchased from the American Type Culture Collection. The cells were cultured in RPMI-1640 medium supplemented with 10% fetal bovine serum (FBS), 2 mM glutamine, 100 U/mL penicillin, 100 μg/mL streptomycin, and 0.05 mM β-mercaptoethanol. The THP-1 cells were not differentiated into macrophage-like cells. The cell density was strictly kept between 0.1–2 × 10^6^ cells/mL. The autophagy reporter cells RAW-Difluo mLC3 expressing the RFP::GFP::LC3 fusion protein to enable detection of autophagic flux were purchased from InvivoGen (Toulouse, France). The cells were cultured in 1x DMEM (Dulbecco’s Modified Eagle Medium), 4.5 g/L glucose, 4 mM L-glutamine, 10% FBS, 100 U/mL penicillin, 100 μg/mL streptomycin, 100 µg/mL Zeocin^TM^. Cells were passaged when they reached a confluency of approx. 80%.

### Cytotoxicity assessment

The toxicity of Au-NPs to THP.1 cells or RAW-Difluo mLC3 cells was determined using the Alamar Blue Cell Viability Assay (Thermo Fischer). To this end, undifferentiated THP.1 cells or RAW-Difluo mLC3 cells were seeded into a 96-well plate at a density of 1 × 10^6^ cells/mL. RAW-Difluo mLC3 cells were seeded 24 h prior to exposure to Au-NPs in order to allow the cells to adhere first. Suspensions of Au-NPs were freshly prepared and used for exposure at the following working concentrations: 0.78, 1.56, 3.13, 6.25, 12.5, 25, 50, 100 µg/mL, in 100 µL complete cell culture medium supplemented with 10% FBS. For the experiments in the presence of inhibitors, cells were exposed 1 h prior and during NP exposure to 3-MA, wortmannin, Mdivi-1, zVAD-fmk, Nec-1, CA-074-ME, Fer-1, and cytochalasin D at 1 mM, 1 µM, 20 µM, 20 µM, 40 µM, 10 µM, 5 µM and 10 µM, respectively. Dimethyl sulfoxide (DMSO) 5% or 4 µM STS, LPS 100 ng/mL, and culture medium alone were used as positive controls for cell death or immune activation and as a negative control, respectively. After exposure, cytotoxic effects were evaluated by incubating with Alamar Blue reagent for 4 h at 37 °C. The resulting fluorescence was read at 540/590 nm (ex/em), using an Infinite 200 Tecan microplate reader operating with Magellan v7.2 software.

### Cellular uptake/localization

THP-1 cells were exposed for 4 h to freshly dispersed Au-NPs at a final concentration of 50 µg/mL. After exposure, the cells were fixed in 2.5% glutaraldehyde in 0.1 M phosphate buffer, pH 7.4 at room temperature for 30 min and further fixed overnight in the refrigerator. Samples were rinsed in 0.1 M phosphate buffer and centrifuged. The pellets were then post-fixed in 2% osmium tetroxide in 0.1 M phosphate buffer, pH 7.4 at 4 °C for 2 h, dehydrated in ethanol followed by acetone and embedded in LX-112. Ultrathin sections (approx. 50–60 nm) were cut by using a Leica ultracut UCT/Leica EM UC 6. Sections were contrasted with uranyl acetate followed by lead citrate and examined using a Tecnai 12 Spirit Bio TWIN transmission electron microscope (FEI Company) at 100 kV/Hitachi HT 7700. Digital images were taken using a Veleta camera (Olympus Soft Imaging Solutions).

### Confocal imaging

THP-1 cells were exposed for 4 h to freshly dispersed Au-5/20-NR3+ NPs at a final concentration of 50 µg/mL. After exposure, the cells were stained with MitoTracker Red CMXRos (Invitrogen) according to the manufacturer instruction and fixed in 3% paraformaldehyde in phosphate buffer at room temperature for 20 min. Glass slides were prepared by cytospin centrifugation and mounted with ProLong Gold Antifade Reagent with DAPI (Invitrogen). Representative images were obtained under a Zeiss LSM 510 confocal microscope equipped with a laser diode 405 nm and HeNe1 543 nm. For the monitoring of autophagy, RAW-Difluo mLC3 cells seeded in a 24-well plate at a density of 0.5 × 10^6^ cells/mL and then exposed to 25 µM rapamycin (Sigma-Aldrich) or 25 µg/mL Au-5/20-NR3+ NPs for 24 h. After washing with PBS cells, were fixed in 4% paraformaldehyde and mounted on glass slides using VECTASHIELD Antifade Mounting Medium with DAPI. Cell imaging was performed using a Zeiss LSM880 confocal microscope equipped with a laser diode 405 nm, argon laser 488 nm and HeNe1 543 nm.

### Mitochondrial function

*Mitochondrial respiration*. Oxygen consumption by living cells^78^ was measured using an Oxygraph instrument (Hansatech Instruments, Norfolk, UK). Briefly, THP-1 cells were exposed to all Au-NPs at 25 µg/mL for 24 h. Cell culture medium alone and STS (4 µM) were used as negative and positive control for cell death, respectively. CCCP (5 µM) was used to drop the mitochondrial membrane potential in order to stimulate the respiration. *Superoxide production*. The production of superoxide was quantified by flow cytometry using the MitoSOX assay (Invitrogen) according to the manufacturer’s instruction. Briefly, the THP-1 cells were exposed to Au-NPs at 25 µg/mL for 24 h. Cell culture medium alone and STS (4 µM) were used as negative and positive control for cell death, respectively. Antimycin A (1 µM), an inhibitor of oxidative phosphorylation, was used as a positive control for superoxide production. *Membrane potential measurements*. Mitochondrial membrane potential was quantified by flow cytometry using the TMRE (tetramethylrhodamine, ethyl ester) assay (Abcam). TMRE is a cell-permeable fluorescent dye that is readily sequestered by active mitochondria. Briefly, the THP-1 cells were exposed to Au-NPs or at 25 µg/mL for 24 h. Cell culture medium alone and STS (4 µM) were used as negative and positive control for cell death, respectively. CCCP (5 µM) was used to uncouple the proton gradient of the electron transport chain in order to drop the mitochondrial membrane potential. *ATP levels*. Total ATP levels were quantified by using the luminescent ATP detection kit (Abcam). Briefly, THP-1 cells were exposed to Au-5/20-NR3+ NPs at the indicated concentrations for 6, 12, and 24 h. Then, cells were lysed, exposed to the ATP substrate solution and the signal measured on a Tecan microplate reader.

### Apoptosis assays

#### Cell cycle/apoptosis assay

For cell cycle distribution analysis, flow cytometry was performed on THP-1 cells exposed for 24 h to Au-5/20-NR3+ NPs at 25 µg/mL. STS (4 µM) was used as positive control for cell death. After treatment, the cells were harvested and fixed with 70% ethanol overnight. The fixed cells were then incubated with RNAse A (1 mg/mL) and propidium iodide (40 µg/mL) for 48 h in the dark at 4 °C. Finally, the DNA content of the cells was analyzed on a BD LSRFortessa cell analyzer operating with FCS Express 4 Flow software. *Caspase activity*. THP-1 cells were exposed for the indicated time-points to Au-5/20-NR3+ NPs at 12.5, 25, and 50 µg/mL. The cells were pre-incubated or not with the pan-caspase inhibitor, zVAD-fmk (10 µM) for 30 min followed by incubation with the NPs. STS (4 µM) was used as positive control for cell death. Caspase-3-like activity was monitored by real-time detection of enzyme-catalyzed AMC release resulting from the cleavage of DEVD-AMC (Molecular Probes), as described previously^79^.

### *C*. *elegans* strains and culture

*Caenorhabditis elegans* N2 (wild-type), *lgg-1(bp500)*, *clp-1(tm690)*, and *ced-3(n2433)* strains used in this work were provided by *Caenorhabditis* Genetics Center (CGC) at the University of Minnesota (Minneapolis, MN) and maintained at 20 °C on nematode growth medium (NGM) plates seeded with *E*. *coli* strain OP50 as described^80^. *E*. *coli* OP50 was cultured overnight in sterilized Luria-Bertani (LB) medium before being spotted on NGM plates. For the toxicity assays, larval stage 4 (L4) worms were incubated with Au-COOH or Au-NH_3_^+^ NPs in 100 μL of M9 buffer (22 mM KH_2_PO_4_, 42 mM Na_2_PO_4_, and 86 mM NaCl) at 0, 5, 10, 50,100, 250, and 500 μg/mL and stirred at 100 rpm for 24 h. Before treatment, *E*. *coli* OP50 and NPs were mixed and sonicated in a water bath sonicator for 5 min. The survival rate of L4 animals (25 animals/assay) was scored 24 h post treatment.

### Transcriptomics analysis

THP-1 cells were exposed for 6 h to Au-NPs with different surface modifications at 4, 15 or 27 µg/mL followed by RNA extraction. Cells incubated with culture medium alone were used as a negative control. After exposure, total RNA was extracted by using the AllPrep DNA/RNA/miRNA Universal Kit (Qiagen). Quality control was performed by NanoDrop (Thermo Scientific) spectrophotometric measurements as well as Qbit fluorometer (Invitrogen) and Bioanalyzer (Agilent) analysis. Sequencing libraries were prepared according to an improved protocol for STRT^81^ adjusted for 10 ng samples by decreasing the number of cycles to 10 during the first PCR amplification. Sequencing was performed using Illumina’s HiSeq2000 sequencing system. The sequencing results were applied to STRTprep^82^ (branch v3dev, commit b866538) for quality assessment, alignment, quantitation and the downstream analysis. In brief, the alignment to human genome UCSC hg19, ERCC spike-ins, ribosomal DNA unit [GenBank: U13356] was by TopHat2^83^, the quantitation was based on NCBI RefSeq gene model, and the differential expression test used was SAMstr.t^84^ Hierarchical clustering of the samples and of the DEGs was applied. The DEG had q-values ≤ 0.05 on multiclass comparison by Kruskal-Wallis with multiple Poisson resampling, corrected by the Storey and Tibshirani method, and p-values ≤ 0.05 on fluctuation comparing to the variation of sequenced spike-in levels, corrected by the Benjamini and Hochberg method. The hierarchical clustering was by Ward’s method with spearman-correlation based distance on the spike-in based normalized levels.

### Proteomics analysis

THP-1 cells were exposed for 24 h to Au-NPs with different surface modifications at 15, 25 or 35 µg/mL followed by protein extraction. STS (4 µM), LPS (100 ng/mL), and culture medium were used as positive controls for cell death and immune activation, and as a negative control, respectively. After exposure, cell pellets were prepared and proteins were extracted, digested and analyzed using a nanoflow HPLC combined with Q Exactive Plus Hybrid Quadrupole-Orbitrap mass spectrometer. Data processing was done using Raw2MGF and ClusterMGF from the Quanti workflow^85^. The data was searched against the human complete proteome database (www.uniprot.org) (downloaded April 2013) using the Mascot search engine (v. 2.5.1) and quantified by Quanti, a software developed in-house for label-free quantification. Student’s t-test, adjusted for multiple testing using false discovery rate (FDR), was used to compare the different treatments on cell proteomes.

### Bioinformatics analysis

Gene Ontology (GO) enrichment analysis was performed by using the GO database and the FunRich functional enrichment analysis tool (version 3.1.3)^86^. The outputs were filtered by adjusted p-value < 0.05 and activation Z-score > 2 or <−2. Comparative causal networks pathway analysis was performed using the Ingenuity Pathway Analysis (IPA) software (IPA; Ingenuity Systems, Redwood City, CA, www.ingenuity.com – version 33559992). Fold-enrichment was considered significant if >1.5. The significance of the pathways was estimated through the curated Ingenuity knowledge database^87^. Known and inferred protein interactions and relationships were considered as a reference set for p-value calculations. The outputs were filtered by p-value < 0.005 and activation Z-score > 2 or <−2. Data were integrated using hierarchical clustering on quantile-normalized data. Values reported in the heatmaps are normalized scores of the -log(p-value) with a p-value < 0.00001 for at least one cluster. Complete linkage and Euclidean distances were employed as metrics to draw association dendrograms. Statistics were performed using R^88^.

### Statistics

Experiments were performed in at least three biological replicates and technical triplicates. Data shown are average values ± S.D. Statistical analysis was performed by one-way ANOVA using Prism 5.02 (GraphPad Software, Inc.), assuming equal variances with P < 0.05.

## Supplementary information

Supporting Information

## Data Availability

The STRT RNA-seq data generated here have been deposited at ArrayExpress (accession no.: E-MTAB-7093) and the proteomics data were submitted to the ProteomeXchange consortium *via* the PRIDE repository^89^ (accession no.: PXD010858).
