# Supplementary material for: Cationic gold nanoparticles elicit mitochondrial dysfunction: a multi-omics study
Source: Sci Rep. 2019 Mar 13;9:4366. doi: 10.1038/s41598-019-40579-6 (PMC6416392; doi:10.1038/s41598-019-40579-6)
Supplement: Supplementary file 1 — Supporting Information [file 41598_2019_40579_MOESM1_ESM.pdf]

SUPPORTING INFORMATION

Cationic gold nanoparticles elicit mitochondrial dysfunction: a multi-omics study

Audrey Gallud[1], Katharina Klöditz[1], Jimmy Ytterberg[2], Nataliya Östberg[2], Shintaro Katayama[3], Tiina Skoog[3], Vladimir Gogvadze[4], Yu-Zen Chen[5], Ding Xue[5], Sergio Moya[6], Jaime Ruiz[7], Didier Astruc[7], Roman Zubarev[2], Juha Kere[3], and Bengt Fadeel[1,*]

*[1]Nanosafety & Nanomedicine Laboratory, Division of Molecular Toxicology, Institute of Environmental Medicine, Karolinska Institutet, 171 77 Stockholm, Sweden; [2]Department of Medical Biochemistry & Biophysics, Karolinska Institutet, 171 77 Stockholm, Sweden; [3]Department of Biosciences & Nutrition, Karolinska Institutet, 141 83 Huddinge, Sweden; [4]Division of Toxicology, Institute of Environmental Medicine, Karolinska Institutet, 171 77 Stockholm, Sweden; [5]Department of Molecular, Cellular, and Developmental Biology, University of Colorado, Boulder, CO 80309, USA; [6]CICbiomaGUNE, 20009 San Sebastian, Spain; [7]ISM, UMR CNRS 5255, Université de Bordeaux, 33405 Talence, France. *Corresponding author: bengt.fadeel@ki.se*

TABLE OF CONTENTS

Supplementary Figure S1. Transcriptomics: hierarchical cluster analysis.

Supplementary Figure S2. Transcriptomics: mitochondrial ox-phos genes.

Supplementary Figure S3. Proteomics data: hierarchical cluster analysis.

Supplementary Figure S4. Caspase activation in cells exposed to Au-NPs.

Supplementary Figure S5. Prolonged exposure of cells to cationic Au-NPs.

Supplementary Table S1. Physicochemical properties of Au nanoparticles.

Supplementary Table S2. GO enrichment analysis of the RNA-seq results.

Supplementary Table S3. Pathway analysis of transcriptomics data by IPA.

FIGURE LEGENDS

Figure S1. Transcriptomics analysis. RNA samples were prepared from THP-1 cells exposed to Au-NPs at 4, 15, or 27 µg/mL and differentially expressed genes (DEGs) were monitored using single-cell tagged reverse transcription (STRT)-RNA sequencing as described in Methods. The figure shows the clustering of expression profile on 777 DEGs over 13 classes of 37 qualified samples. The gradation (z-score) indicates the direction of up- or down-regulated genes normalized for expression level, per gene, among samples in the heatmap. Based on the hierarchical clustering, the DEGs were grouped into three clusters designated I, II, and III. The clusters were subjected to pathway analysis as reported in the main text (and see Table S2, S3).

Figure S2. Dysregulation of multiple genes belonging to the oxidative phosphorylation and mitochondrial dysfunction pathways. THP-1 cells were exposed to Au-5/20-NH3+ NPs (15 µg/mL) for 6 h prior to RNA-seq. For a graphical depiction of dysregulated ox-phos genes, see Figure 4A.

Figure S3. Proteomics analysis. Proteins were extracted from THP-1 cells exposed to Au-NPs of two different sizes at 15, 25 or 35 µg/mL for 24 h and expression changes were monitored using mass spectrometry-based protocols. Note that hierarchical clustering analysis showed that the ammonium-modified Au-NPs (5 nm and 20 nm) were clustered together. LPS (0.1 µg/mL) and STS (4 µM) were used as controls for cell activation/inflammation and cell death, respectively.

Figure S4. Caspase-3-like activity was monitored by real-time detection of caspase-catalyzed cleavage of DEVD-AMC. No caspase activity was seen in control cells (A). STS (4 µM) was used as positive control for apoptosis and cells were pre-incubated with the pan-caspase inhibitor, zVAD-fmk (10 µM) for 30 min to confirm that AMC release was caspase-dependent (B). THP-1 cells were exposed for 4 h to Au-5-NR3+ NPs (C) and Au-20-NR3+ NPs (D) at the indicated concentrations. Data shown are means of triplicate samples from two experiments ± S.D.

Figure S5. Effects of prolonged exposure to cationic Au-NPs. (A) THP-1 cells were exposed to 25 µg/mL or 50 µg/mL Au-5-NR3+ NPs or Au-20-NR3+ NPs for the indicated time-points. The mitochondrial uncoupling agent, CCCP (100 µM) was used as a positive control. Cellular ATP levels were measured by using the CellTiter-Glo2.0 assay (Promega). Data are mean values of quadruplicate samples from two independent experiments ± S.D. THP-1 cells were exposed to 25 µg/mL of Au-5-NR3+ NPs (B) and Au-20-NR3+ NPs (C) for the indicated time-points, and caspase-3-like activity was monitored by real-time detection of caspase-catalyzed AMC release. STS (4 µM) was used as positive control for apoptosis. Following a time-dependent increase in caspase activation, caspase activation was no longer seen at 48 h, in line with ATP results. Data in (B) and (C) are mean values of triplicate samples from two independent experiments ± S.D.

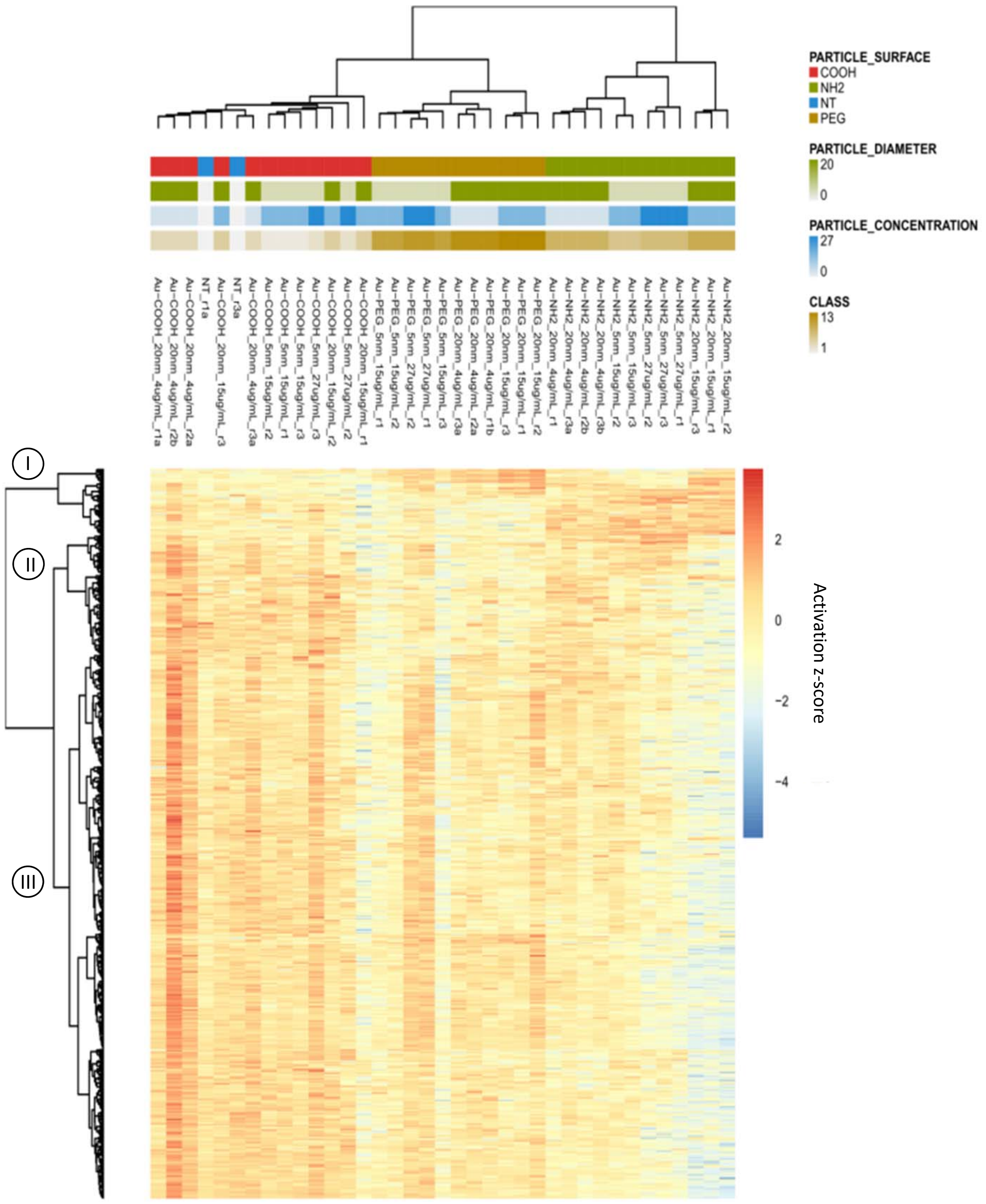
PARTICLE_SURFACE
COOH
NH2
NT
PEG
PARTICLE_DIAMETER
20
0
PARTICLE_CONCENTRATION
27
0
CLASS
13
1
Au-COOH_20nm_4ug/mL_r1a
Au-COOH_20nm_4ug/mL_r2b
Au-COOH_20nm_4ug/mL_r2a
NT_r1a
Au-COOH_20nm_15ug/mL_r3
NT_r3a
Au-COOH_20nm_4ug/mL_r3a
Au-COOH_5nm_15ug/mL_r2
Au-COOH_5nm_15ug/mL_r1
Au-COOH_5nm_15ug/mL_r3
Au-COOH_5nm_27ug/mL_r3
Au-COOH_20nm_15ug/mL_r2
Au-COOH_5nm_27ug/mL_r2
Au-COOH_20nm_15ug/mL_r1
Au-PEG_5nm_15ug/mL_r1
Au-PEG_5nm_15ug/mL_r2
Au-PEG_5nm_27ug/mL_r2
Au-PEG_5nm_27ug/mL_r1
Au-PEG_5nm_15ug/mL_r3
Au-PEG_20nm_4ug/mL_r3a
Au-PEG_20nm_4ug/mL_r2a
Au-PEG_20nm_4ug/mL_r1b
Au-PEG_20nm_15ug/mL_r3
Au-PEG_20nm_15ug/mL_r1
Au-PEG_20nm_15ug/mL_r2
Au-NH2_20nm_4ug/mL_r1
Au-NH2_20nm_4ug/mL_r3a
Au-NH2_20nm_4ug/mL_r2b
Au-NH2_20nm_4ug/mL_r3b
Au-NH2_5nm_15ug/mL_r2
Au-NH2_5nm_15ug/mL_r3
Au-NH2_5nm_27ug/mL_r2
Au-NH2_5nm_27ug/mL_r3
Au-NH2_5nm_27ug/mL_r1
Au-NH2_20nm_15ug/mL_r3
Au-NH2_20nm_15ug/mL_r1
Au-NH2_20nm_15ug/mL_r2
I
II
III
2
0
-2
-4
Activation z-score

Figure S1

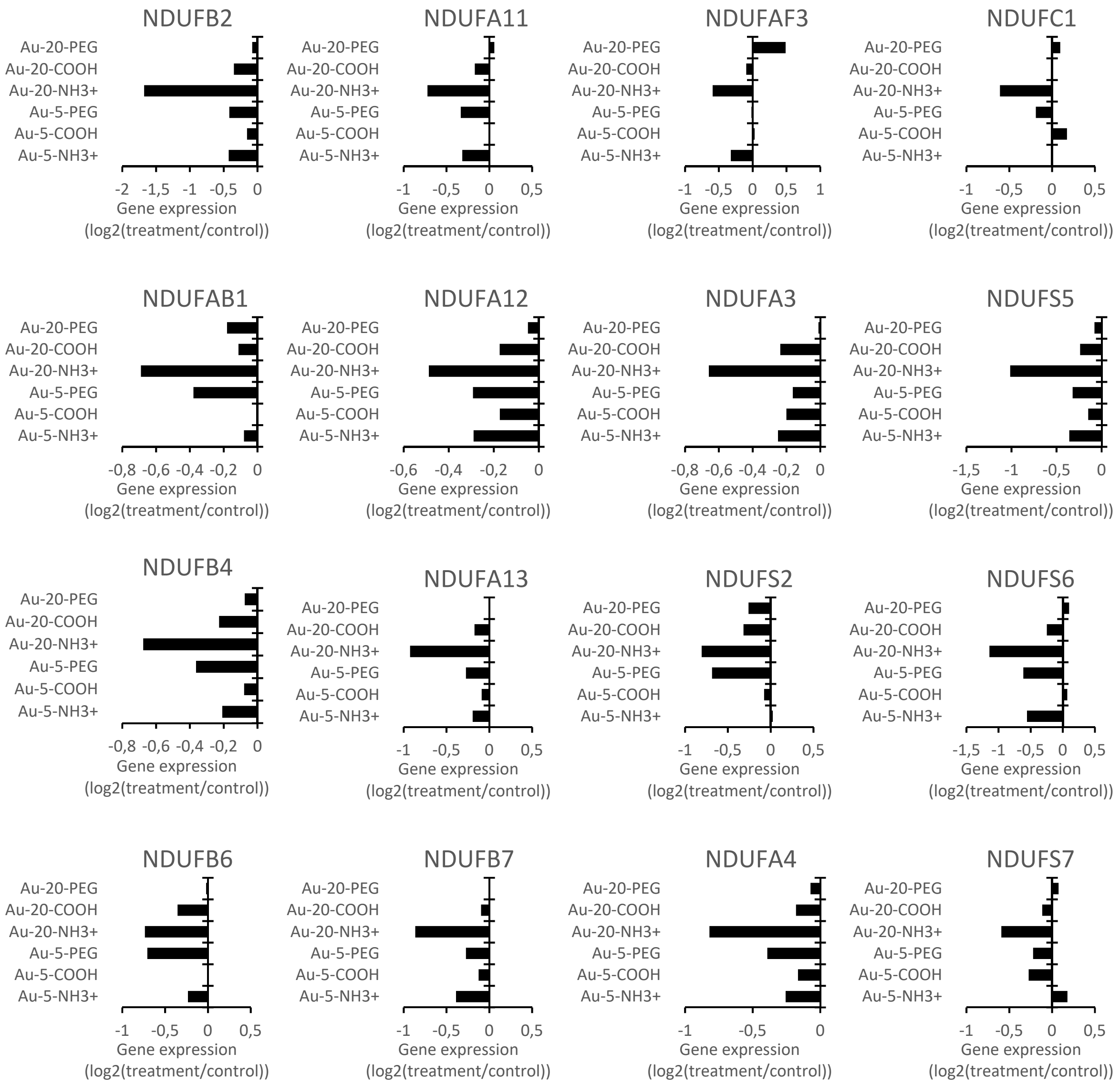
NDUFB2
Au-20-PEG
Au-20-COOH
Au-20-NH3+
Au-5-PEG
Au-5-COOH
Au-5-NH3+
-2 -1,5 -1 -0,5 0
Gene expression
(log2(treatment/control))
NDUFA11
Au-20-PEG
Au-20-COOH
Au-20-NH3+
Au-5-PEG
Au-5-COOH
Au-5-NH3+
-1 -0,5 0 0,5
Gene expression
(log2(treatment/control))
NDUFAF3
Au-20-PEG
Au-20-COOH
Au-20-NH3+
Au-5-PEG
Au-5-COOH
Au-5-NH3+
-1 -0,5 0 0,5 1
Gene expression
(log2(treatment/control))
NDUFC1
Au-20-PEG
Au-20-COOH
Au-20-NH3+
Au-5-PEG
Au-5-COOH
Au-5-NH3+
-1 -0,5 0 0,5
Gene expression
(log2(treatment/control))
NDUFAB1
Au-20-PEG
Au-20-COOH
Au-20-NH3+
Au-5-PEG
Au-5-COOH
Au-5-NH3+
-0,8 -0,6 -0,4 -0,2 0
Gene expression
(log2(treatment/control))
NDUFA12
Au-20-PEG
Au-20-COOH
Au-20-NH3+
Au-5-PEG
Au-5-COOH
Au-5-NH3+
-0,6 -0,4 -0,2 0
Gene expression
(log2(treatment/control))
NDUFA3
Au-20-PEG
Au-20-COOH
Au-20-NH3+
Au-5-PEG
Au-5-COOH
Au-5-NH3+
-0,8 -0,6 -0,4 -0,2 0
Gene expression
(log2(treatment/control))
NDUFS5
Au-20-PEG
Au-20-COOH
Au-20-NH3+
Au-5-PEG
Au-5-COOH
Au-5-NH3+
-1,5 -1 -0,5 0
Gene expression
(log2(treatment/control))
NDUFB4
Au-20-PEG
Au-20-COOH
Au-20-NH3+
Au-5-PEG
Au-5-COOH
Au-5-NH3+
-0,8 -0,6 -0,4 -0,2 0
Gene expression
(log2(treatment/control))
NDUFA13
Au-20-PEG
Au-20-COOH
Au-20-NH3+
Au-5-PEG
Au-5-COOH
Au-5-NH3+
-1 -0,5 0 0,5
Gene expression
(log2(treatment/control))
NDUFS2
Au-20-PEG
Au-20-COOH
Au-20-NH3+
Au-5-PEG
Au-5-COOH
Au-5-NH3+
-1 -0,5 0 0,5
Gene expression
(log2(treatment/control))
NDUFS6
Au-20-PEG
Au-20-COOH
Au-20-NH3+
Au-5-PEG
Au-5-COOH
Au-5-NH3+
-1,5 -1 -0,5 0 0,5
Gene expression
(log2(treatment/control))
NDUFB6
Au-20-PEG
Au-20-COOH
Au-20-NH3+
Au-5-PEG
Au-5-COOH
Au-5-NH3+
-1 -0,5 0 0,5
Gene expression
(log2(treatment/control))
NDUFB7
Au-20-PEG
Au-20-COOH
Au-20-NH3+
Au-5-PEG
Au-5-COOH
Au-5-NH3+
-1 -0,5 0 0,5
Gene expression
(log2(treatment/control))
NDUFA4
Au-20-PEG
Au-20-COOH
Au-20-NH3+
Au-5-PEG
Au-5-COOH
Au-5-NH3+
-1 -0,5 0
Gene expression
(log2(treatment/control))
NDUFS7
Au-20-PEG
Au-20-COOH
Au-20-NH3+
Au-5-PEG
Au-5-COOH
Au-5-NH3+
-1 -0,5 0 0,5
Gene expression
(log2(treatment/control))

Figure S2

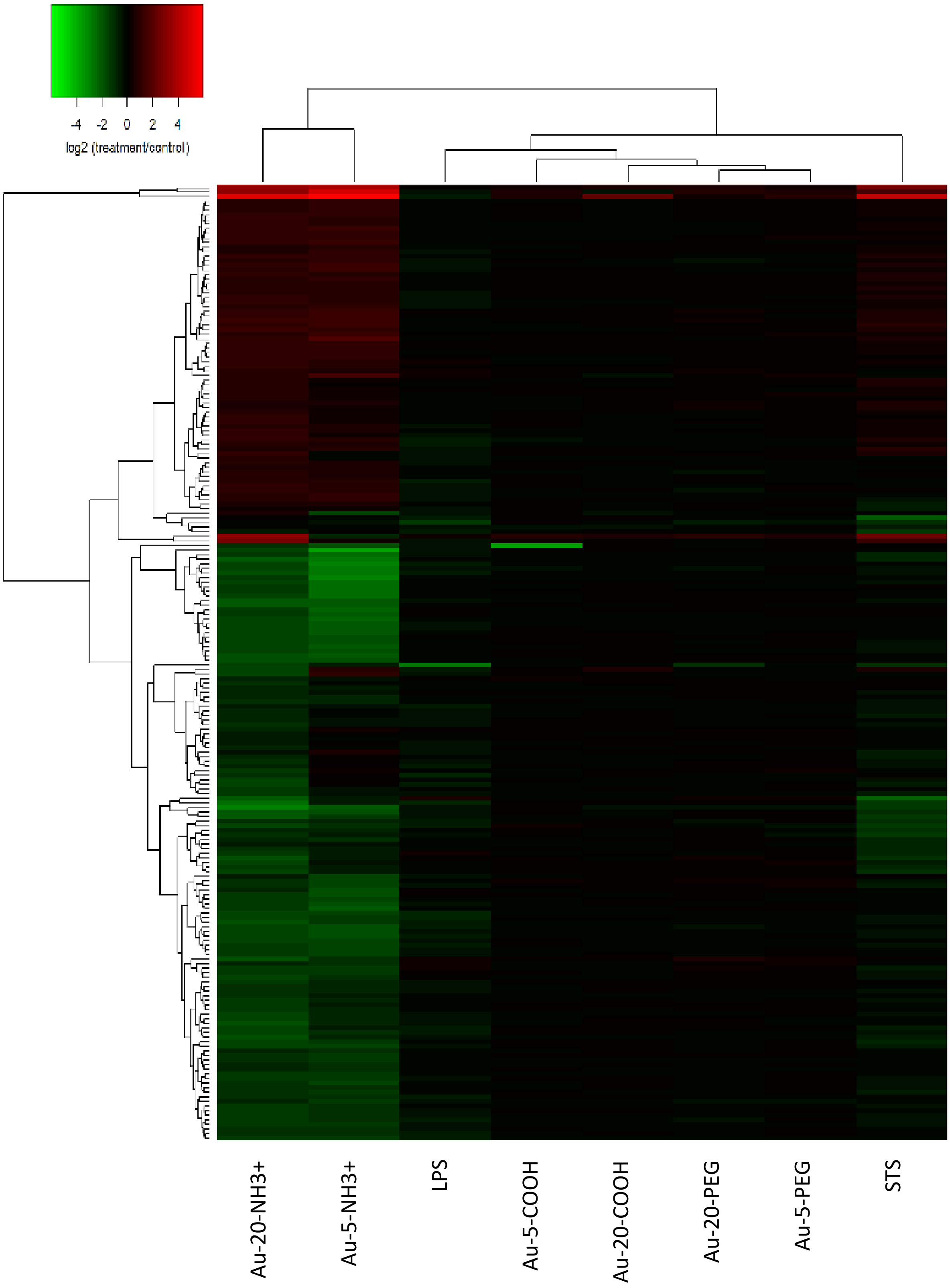

-4 -2 0 2 4
log2 (treatment/control)
Au-20-NH3+
Au-5-NH3+
LPS
Au-5-COOH
Au-20-COOH
Au-20-PEG
Au-5-PEG
STS

Figure S3

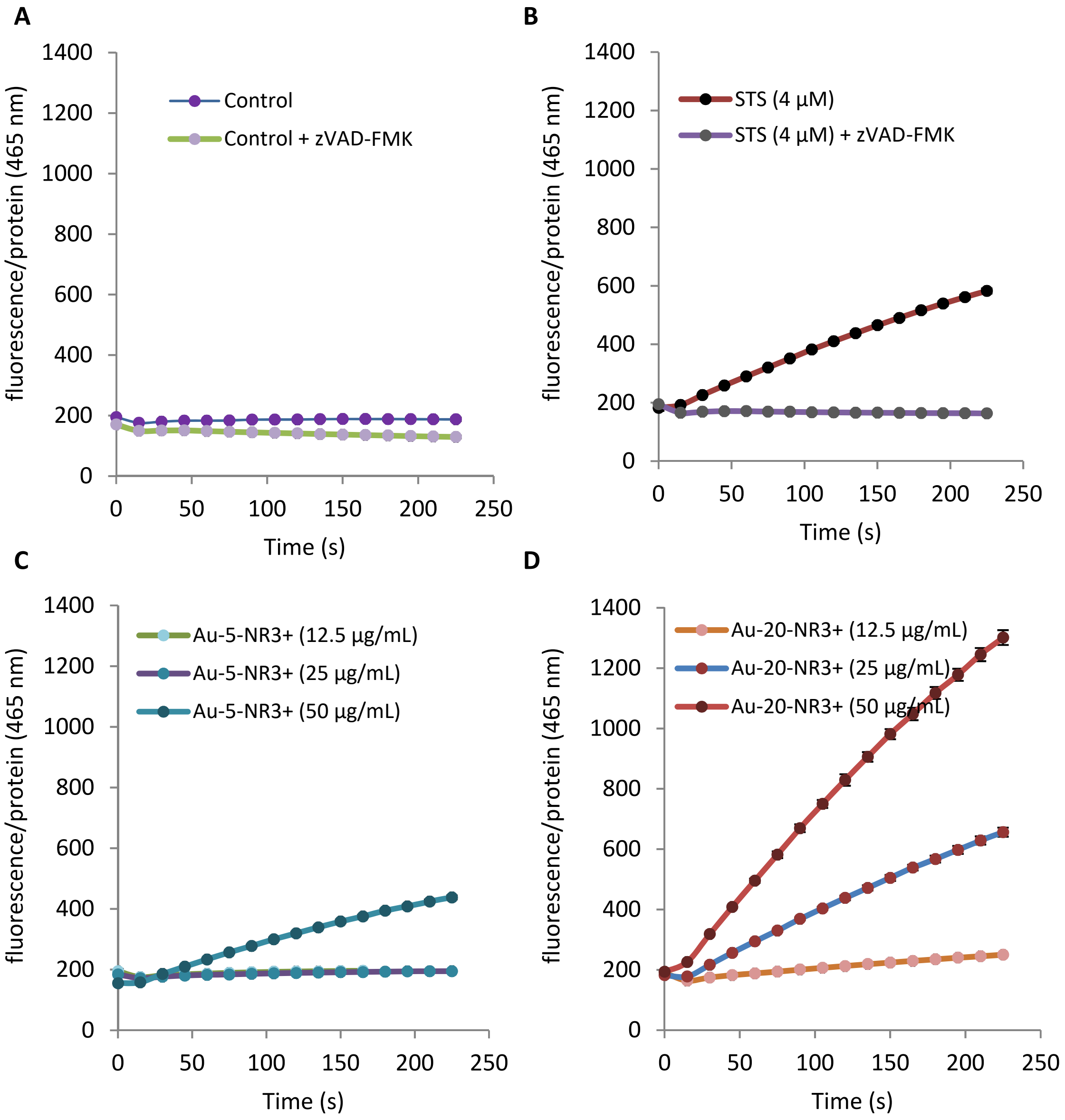
A
B
C
D
fluorescence/protein (465 nm)
Time (s)
Control
Control + zVAD-FMK
STS (4 µM)
STS (4 µM) + zVAD-FMK
Au-5-NR3+ (12.5 µg/mL)
Au-5-NR3+ (25 µg/mL)
Au-5-NR3+ (50 µg/mL)
Au-20-NR3+ (12.5 µg/mL)
Au-20-NR3+ (25 µg/mL)
Au-20-NR3+ (50 µg/mL)
0
200
400
600
800
1000
1200
1400
0
50
100
150
200
250

Figure S4

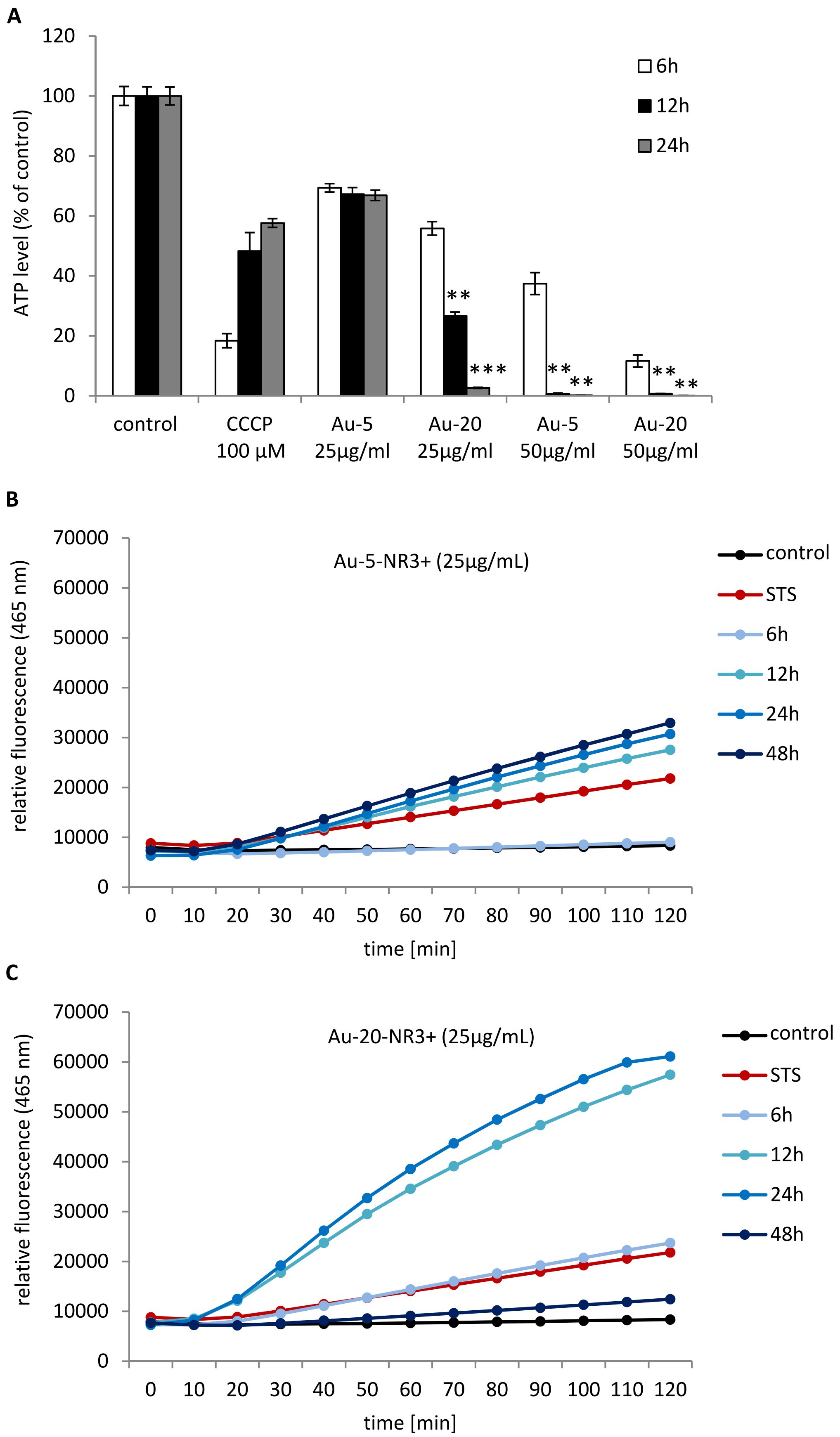
A
ATP level (% of control)
120
100
80
60
40
20
0
6h
12h
24h
**
***
**
**
**
**
control
CCCP 100 µM
Au-5 25µg/ml
Au-20 25µg/ml
Au-5 50µg/ml
Au-20 50µg/ml
B
relative fluorescence (465 nm)
70000
60000
50000
40000
30000
20000
10000
0
Au-5-NR3+ (25µg/mL)
control
STS
6h
12h
24h
48h
0 10 20 30 40 50 60 70 80 90 100 110 120
time [min]
C
relative fluorescence (465 nm)
70000
60000
50000
40000
30000
20000
10000
0
Au-20-NR3+ (25µg/mL)
control
STS
6h
12h
24h
48h
0 10 20 30 40 50 60 70 80 90 100 110 120
time [min]

Figure S5

Particle	Surface chemistry	Size measurements: TEM	DLS (in water)		DLS (in culture media with serum)		zeta-potential (in water) (mV)	zeta-potential (in culture media with serum) (mV)	UV-vis (in water)
		Primary size (nm)	Z-Ave d. (nm)	PdI	Z-Ave d. (nm)	PdI			Absorption band λ_{max} (nm)
Au-5-NR3+	$-S(CH_2)_{11}N(CH_3)_3Br$ and $S(CH_2)_{11}CH_3$	2.5 ± 1.5	47 ± 1	0.544 ± 0.002	96 ± 14	0.570 ± 0.002	19.90 ± 0.88	-7.05 ± 0.18	516
Au-5-COOH	$-S(CH_2)_{10}CO_2Na$	4.5 ± 1.5	77 ± 6	0.434 ± 0.068	135 ± 1	0.687 ± 0.024	-30.63 ± 1.27	-6.98 ± 0.37	528
Au-5-PEG	$-SPEG_{550}$	3.5 ± 1.2	32 ± 1	0.561 ± 0.020	31 ± 1	0.486 ± 0.025	-4.01 ± 0.75	-4.78 ± 0.50	520
Au-20-NR3+	$-S(CH_2)_{11}N(CH_3)_3Br$	15.0 ± 5.0 and 46.0 ± 10.0	236 ± 23	0.183 ± 0.009	312 ± 6	0.271 ± 0.006	17.90 ± 0.37	-9.35 ± 0.61	530
Au-20-COOH	$-S(CH_2)_{10}CH_2O(C_2H_4O)_3C_2H_4OCH_2CO_2Na$	14.0 ± 3.5	30 ± 1	0.445 ± 0.018	37 ± 1	0.483 ± 0.009	-21.23 ± 0.45	-8.21 ± 0.95	529
Au-20-PEG	$-SPEG_{550}$	13.0 ± 3.0	20 ± 1	0.161 ± 0.008	23 ± 1	0.262 ± 0.004	-5.92 ± 1.13	-6.40 ± 0.57	524

Table S1. Physical-chemical properties of Au-NPs. Primary sizes and sizes of Au-NPs in suspension were determined by using transmission electron microscopy (TEM), dynamic light scattering (DLS) coupled with zeta potential measurements. The maximum absorption band (λ_{max}) was measured by UV-vis spectroscopy.

Cluster I	P-value	Adjusted P-value	Z-score
cellular response to biotic stimulus (GO:0071216)	9,5E-10	1,3E-06	-2,32
lipopolysaccharide-mediated signaling pathway (GO:0031663)	5,4E-08	1,5E-05	-2,66
cellular response to molecule of bacterial origin (GO:0071219)	7,9E-09	3,7E-06	-2,32
cellular response to lipopolysaccharide (GO:0071222)	5,2E-09	3,6E-06	-2,30
positive regulation of leukocyte migration (GO:0002687)	1,1E-08	4,0E-06	-2,27
granulocyte activation (GO:0036230)	8,3E-07	1,1E-04	-2,87
inflammatory response (GO:0006954)	8,4E-08	2,0E-05	-2,37
response to lipopolysaccharide (GO:0032496)	1,1E-07	2,2E-05	-2,39
response to molecule of bacterial origin (GO:0002237)	1,9E-07	3,0E-05	-2,42
regulation of leukocyte migration (GO:0002685)	1,2E-07	2,2E-05	-2,31

Cluster II	P-value	Adjusted P-value	Z-score
mRNA processing (GO:0006397)	1,5E-06	0,0022	-2,39
RNA splicing (GO:0008380)	5,1E-06	0,0025	-2,34
protein polymerization (GO:0051258)	3,2E-06	0,0023	-2,26
'de novo' posttranslational protein folding (GO:0051084)	1,5E-05	0,0053	-2,25
'de novo' protein folding (GO:0006458)	2,4E-05	0,0068	-2,21
posttranscriptional regulation of gene expression (GO:0010608)	1,1E-04	0,0204	-2,45
microtubule-based process (GO:0007017)	1,1E-04	0,0204	-2,42
mitotic sister chromatid segregation (GO:0000070)	2,6E-04	0,0348	-2,66
negative regulation of protein modification by small protein conjugation or removal (GO:1903321)	1,1E-04	0,0204	-2,21
microtubule cytoskeleton organization (GO:0000226)	1,7E-04	0,0280	-2,38

Cluster III	P-value	Adjusted P-value	Z-score
respiratory electron transport chain (GO:0022904)	2,0E-38	5,5E-35	-2,17
electron transport chain (GO:0022900)	7,9E-38	1,1E-34	-2,15
gene expression (GO:0010467)	3,3E-34	3,0E-31	-2,33
RNA splicing (GO:0008380)	2,4E-28	1,7E-25	-2,33
translation (GO:0006412)	1,4E-26	7,8E-24	-2,35
generation of precursor metabolites and energy (GO:0006091)	6,7E-26	3,1E-23	-2,36
RNA splicing, via transesterification reactions with bulged adenosine as nucleophile (GO:0000377)	1,6E-25	5,4E-23	-2,21
mRNA splicing, via spliceosome (GO:0000398)	1,6E-25	5,4E-23	-2,21
RNA splicing, via transesterification reactions (GO:0000375)	7,8E-25	2,4E-22	-2,21
mRNA processing (GO:0006397)	3,1E-22	8,5E-20	-2,37

Table S2. GO enrichment analysis. Top 10 of the biological process GO terms, sorted by the adjusted p-value. Data show the GO terms by cluster and the corresponding p-value, adjusted p-value and Z-score.

Cluster	Top Canonical Pathways	Category	Probability of association (p-value)
I	Role of Hypercytokinemia / hyperchemokinemia in the Pathogenesis of Influenza	Disease-Specific Pathways; Pathogen-Influenced Signaling	6,94E-11
	Communication between Innate and Adaptative Immune Cells	Cellular Immune Response	1,25E-09
	Differential Regulation of Cytokine Production in Macrophages and T Helper Cells by IL-17A and IL-17F	Cytokine Signaling	9,64E-09
	Agranulocyte Adhesion and Diapedesis	Cellular Immune Response	1,15E-08
	Granulocyte Adhesion and Diapedesis	Cellular Immune Response	1,15E-08
II	Remodeling of Ephithelial Adherens Jnctions	Cellular Growth, Proliferation and Development	1,73E-04
	Sertoli Cell-Sertoli Cell Junction Signaling	Cellular Growth, Proliferation and Development	1,99E-03
	14-3-3-mediated Signaling	Apoptosis; Cell Cycle Regulation	3,12E-03
	Phagosome Maturation	Cellular Immune Response; Pathogen-Influenced Signaling	3,84E-03
	Epithelial Adherens Junction Signaling	Cellular Growth, Proliferation and Development	4,24E-03
III	Oxidative Phosphorylation	Electron Transfer	5,02E-42
	Mitochondrial Dysfunction	Disease-specific pathways	8,37E-39
	Protein Ubiquitination Pathway	Intracellular and second messenger signaling	1,51E-15
	Systemic Lupus Erythematosus Signaling	Disease-specific pathways	1,04E-06
	EIF2 Signaling	Cellular growth, Proliferation and development, Cellular stress and injury, intracellular and second messenger signaling	2,58E-06

Table S3. Pathway analysis. Single core analyses were performed on the list of DEGs classified into 3 clusters by using IPA software (refer to Figure 3 for the 3 clusters). The top 5 canonical pathways, with the corresponding p-value and category, were identified for the 5 individual clusters.
